# Type 1 diabetes genetic risk score variation across ancestries using whole genome sequencing and array-based approaches

**DOI:** 10.1038/s41598-024-82278-x

**Published:** 2024-12-28

**Authors:** Ankit M. Arni, Diane P. Fraser, Seth A. Sharp, Richard A. Oram, Matthew B. Johnson, Michael N. Weedon, Kashyap A. Patel

**Affiliations:** 1 Department of Clinical and Biomedical Sciences, RILD Building, Royal Devon and Exeter Hospital, University of Exeter, Barrack Road, Exeter, EX2 5DW UK; 2 Department of Pediatrics, Stanford University, Stanford, CA 94305 USA

**Keywords:** Type 1 diabetes, Population genetics, Population screening, Genetic testing

## Abstract

A Type 1 Diabetes Genetic Risk Score (T1DGRS) aids diagnosis and prediction of Type 1 Diabetes (T1D). While traditionally derived from imputed array genotypes, Whole Genome Sequencing (WGS) provides a more direct approach and is now increasingly used in clinical and research studies. We investigated the concordance between WGS-based and array-based T1DGRS across genetic ancestries in 149,265 UK Biobank participants using WGS, TOPMed-imputed, and 1000 Genomes-imputed array genotypes. In the overall cohort, WGS-based T1DGRS demonstrated strong correlation with TOPMed-imputed array-based score (*r* = 0.996, average WGS-based score 0.0028 standard deviations (SD) lower, *p* < 10^− 31^), while showing lower correlation with 1000 Genomes-imputed array-based scores (*r* = 0.981, 0.043 SD lower in WGS, *p* < 10^− 300^). Ancestry-stratified analyses between WGS-based and TOPMed-imputed array-based score showed the highest correlation with European ancestry (*r* = 0.996, 0.044 SD lower in WGS, *p* < 10^− 300^) followed by African ancestry (*r* = 0.989, 0.0193 SD lower in WGS, *p* < 10^− 14^) and South Asian ancestry (*r* = 0.986, 0.0129 SD lower in WGS, *p* < 10 ^− 6^). These differences were more pronounced when comparing WGS based score with 1000 Genomes-imputed array-based scores (*r* = 0.982, 0.975, 0.957 for European, South Asian, African respectively). Population-level analysis using WGS-based T1DGRS revealed significant ancestry-based stratification, with European ancestry individuals showing the highest scores, followed by South Asian (average 0.28 SD lower than Europeans, *p* < 10^− 58^) and African ancestry individuals (average 0.89 SD lower than Europeans, *p* < 10^− 300^). Notably, when applying the European ancestry-derived 90^th^ centile risk threshold, only 0.71% (95% CI 0.41–1.13) of African ancestry individuals and 6.4% (95% CI 5.6–7.2) of South Asian individuals were identified as high-risk, substantially below the expected 10%. In conclusion, while WGS is viable for generating T1DGRS, with TOPMed-imputed genotypes offering a cost-effective alternative, the persistence of ancestry-based variations in T1DGRS distribution even using whole genome sequencing emphasises the need for ancestry-specific or pan-ancestry standards in clinical practice.

**Supplementary Information:**

The online version contains supplementary material available at 10.1038/s41598-024-82278-x.

## Introduction

The rapidly expanding clinical applications of the Type 1 Diabetes Genetic Risk Score (T1DGRS) underline the importance of precision in score calculation, to ensure efficacy in diverse clinical and research settings. A T1DGRS provides personalised insight into an individual’s genetic predisposition to T1D by aggregating the individual contributions of multiple genetic variants into a single metric^1^. The T1DGRS has demonstrated valuable clinical utility in distinguishing T1D from Type 2 Diabetes (T2D) in diagnostically uncertain cases^2,3^. It can aid in selecting individuals for monogenic diabetes genetic testing (GRS < 50th centile of a T1D population)^4–6^. More recently, it is also shown to identify individuals with a high-risk of T1D development in the population (GRS ≥ 90^th^ centile of the background population), who can then be targeted for intervention^7^. These clinical applications support the need for an accurate measurement of the T1DGRS.

Short-read whole genome sequencing (WGS) is gaining prominence as a first-line approach for genomic studies. It provides comprehensive coverage of both coding and non-coding regions of the genome, irrespective of genetic ancestry^8,9^. This makes it ideal for generating a GRS, since most risk variants are non-coding^10,11^. However, WGS is expensive, requires substantial computing resources and storage infrastructure, and faces challenges in calling variants within repetitive, extremely GC-rich regions and homopolymer sequences^12^. Array genotyping has been extensively used in generating risk scores including T1DGRS but requires imputation of a large number of unassayed genotypes across the genome based on a reference panel of deep-sequenced individuals^2,3,13^. Imputation can also be challenging for non-European populations, due to underrepresentation of these groups in imputation reference panels^14,15^. In addition to imputation challenges, array genotypes have limited coverage, potential for allelic dropout, susceptibility to probe cross-hybridization, and carries the risk of significant batch effects^16^. Despite the advantages of WGS, it is unclear how WGS-based T1DGRS differs from the widely used array-genotyping based T1DGRS.

We therefore aimed to compare the T1DGRS derived from array genotypes, using two commonly used imputation reference panels, against scores derived from WGS. We used a large multi-ancestry population cohort to provide valuable insights into the suitability and reliability of each method in diverse populations and contribute to better application of the T1DGRS in routine clinical practice.

## Methods

### Study cohort

We used data from the UK Biobank, an ethically approved population cohort of over 500,000 individuals from the UK^17^. The UK Biobank, an observational study, contains detailed genetic information, including WGS and array genotyping data and phenotypic information from self-reports, hospital records, and general practitioners^17^. We focused on a subset of 149,265 individuals matched for having both WGS and array genotyping data, to allow for direct comparison between these two genetic data sources. Of these, 137,888 (92.4%) were of European genetic ancestry, 2,404 (1.6%) of African genetic ancestry, 3,346 (2.2%) of South Asian genetic ancestry and 5,627 (3.8%) of a genetic ancestry other than the above, based on principal component analysis using reference data from the 1000 Genomes Project. We defined ancestry based on genetic similarity to the 1000 Genomes reference panel super-population groups, following the methodology described by Pain et al.^18^. In brief, we derived a multinomial elastic net model predicting super-population membership using the glmnet R package using the first six reference-projected genetic principal components, then projected the reference-derived principal components into the target dataset and applied the reference-derived elastic net model to predict population membership^19,20^. Individuals in the target dataset were assigned to a population when the predicted probability exceeded 0.95.

### Genomic data

#### Whole genome sequencing

We analysed joint variant calling data from the first phase samples, sequenced by deCODE Genetics, for our study^21^. Detailed information on the WGS data can be found at https://biobank.ctsu.ox.ac.uk/showcase/label.cgi?id=180 and is described in depth by Halldorsson et al.^21^. We extracted 67 T1D-associated variants used for generating the T1DGRS^3^. To minimize false positive calls, we only included calls with a read depth (DP) ≥ 10, genotype quality (GQ) ≥ 20, and allelic balance (or variant allelic fraction, VAF) between 0.2 and 0.8 for heterozygous calls^22^.

#### Array genotyping

The UK Biobank samples were SNP-genotyped using two custom-made Affymetrix chips: the UK BiLEVE Axiom for the first 50,000 individuals, and the Affymetrix UK Biobank Axiom array for the remaining participants. These customised genotyping arrays comprised 820,967 single nucleotide variants (SNVs) and short insertions/deletions (INDELs) (https://biobank.ctsu.ox.ac.uk/crystal/label.cgi?id=100319). This directly genotyped data was imputed by UK Biobank using two reference panels.

Initially, they used a combined reference panel of Haplotype Reference Consortium (HRC), UK10K and 1000 Genomes Project Phase 3 panels (henceforth referred to as the 1000 Genomes reference panel). The HRC panel consists of 39,235,157 sites and 32,488 samples, primarily of European ancestry, described in detail in (https://www.sanger.ac.uk/collaboration/haplotype-reference-consortium/)^23^. The UK10K panel includes 3,781 samples, all of European ancestry (https://www.sanger.ac.uk/collaboration/uk10k-project/)^24^. The 1000 Genomes Project Phase 3 panel comprises 2,504 samples: 20% each from European, South Asian, and East Asian ancestries, 14% admixed American, and 26% African ancestry (https://www.internationalgenome.org)^25,26^. The UK10K haplotype reference panel was merged with the 1000 Genomes Phase 3 reference panel using IMPUTE2 software, resulting in 87,696,888 biallelic variants across 12,570 haplotypes. The pre-imputation variant QC, phasing, and imputation conducted on the combined UK Biobank and UK BiLEVE dataset have been described in detail (https://biobank.ctsu.ox.ac.uk/crystal/refer.cgi?id=157020). In brief, phasing was conducted using SHAPEIT3 in chunks of 5,000 variants with a 250-variant overlap between chunks. Imputation was performed using IMPUTE3 in 2 Mb chunks with a 250 kb buffer region.

More recently, UK Biobank released array genotypes imputed using the TOPMed R2 panel, containing 97,256 deeply sequenced human genomes and 308,107,085 genetic variants, of which 55% of individuals were from non-European genetic ancestries (https://imputation.biodatacatalyst.nhlbi.nih.gov/#!)^27,28^. The TOPMed Informatics Research Center performed this analysis centrally (https://biobank.ndph.ox.ac.uk/crystal/field.cgi?id=21007). Briefly, phasing was carried out on 81 chromosomal chunks using Eagle v.2.4. The phased data were converted from GRCh37 to GRCh38 using UCSC LiftOver. Imputation was performed using Minimac4 v1.0.2 (https://genome.sph.umich.edu/wiki/Minimac4) in 1 Mb chunks and merged back together by chromosome. Due to the large data size, markers with poor imputation were not retained (excluded Minimac4 imputation quality metric R^2^ < 0.1).

All variants used to generate the risk scores in this study were well-imputed, having an imputation quality score > 0.9 in both panels (Suppl. Table 1).

#### Type 1 diabetes genetic risk score

We defined individuals with Type 1 Diabetes (T1D) as per Sharp et al. if they met the following criteria: diabetes onset at ≤ 18 years of age, initiation of insulin treatment within one year of diagnosis, and continued insulin use at the time of recruitment. We focused on early-onset cases due to the lower risk of misclassification, which is a significant issue in later-onset T1D, particularly given the lack of islet autoantibody data in the UK Biobank^29–31^. We have provided baseline clinical characteristics in Suppl. Table 2.

Our study aims to assess a previously validated Type 1 Diabetes (T1D) genetic risk score to maximise the immediate impact of our work on current clinical and research studies. We deliberately chose not to generate a new T1DGRS, as an unvalidated score would severely limit the study’s practical implications. Although genome-wide polygenic risk scores (PRS) are gaining popularity, their additive models are not optimal for type 1 diabetes because the largest risk contribution comes from HLA interactions, which account for > 50% of all T1D risk^32,33^. We therefore focused on the most recent iteration of the T1DGRS by Sharp et al., referred to as T1DGRS2 in the original publication^3^. This GRS demonstrated the strongest evidence of clinical utility in the literature for a T1DGRS, widespread adoption in research studies, and modelled interactions between HLA variants, which is crucial for capturing T1D risk. This resulted in exceptional discrimination (ROC AUC 0.92). It is widely used and has evidence of contributing to the progression of type 1 diabetes^34^, classifying type 1 diabetes in multiethnic youth^35^ and predicting T1D onset in the general population^7^. We computed a weighted T1DGRS using the genotypes of 67 T1D-associated variants identified in the Type 1 Diabetes Genetics Consortium (T1DGC) cohort, as described by Sharp et al. (Suppl. Table 1). Sharp et al. performed a genome-wide association study (GWAS) using ImmunoChip case-control genetic data from European ancestry subjects (6,670 T1D cases and 9,416 controls). The 67 variants include 35 HLA and 32 non-HLA variants. Importantly, Sharp et al. modelled interactions between 14 variants marking strongly associated HLA DR-DQ haplotypes and generated odds ratios to create this improved GRS. This substantially enhances its ability to capture T1D risk compared to other GRSs^3^.

We computed a weighted T1DGRS using the genotypes of 67 T1D-associated variants identified in the Type 1 Diabetes Genetics Consortium (T1DGC) cohort, as described by Sharp et al. (Suppl. Table 1). Sharp et al. performed a genome-wide association study (GWAS) using ImmunoChip case-control genetic data from European ancestry subjects (6,670 T1D cases and 9,416 controls). The 67 variants include 35 HLA and 32 non-HLA variants. Importantly, Sharp et al. modelled interactions between 14 variants marking strongly associated HLA DR-DQ haplotypes and generated odds ratios to create this improved GRS. This substantially enhances its ability to capture T1D risk compared to other GRSs^3^.

### Statistical analysis

We applied Spearman’s rank correlation to compare T1DGRS from Whole Genome Sequencing genotypes, from 1000 Genomes-imputed array genotypes, and from TOPMed-imputed array genotypes. Spearman’s rank correlation was selected as it does not assume linearity or normality and is less sensitive to outliers. We employed the correlation z-test by Meng et al. to determine whether the difference between correlation coefficient pairs was significant and is particularly useful when correlations are derived from the same samples^36^. Additionally, we utilised the two-sided paired t-test to evaluate the mean of the differences in T1DGRS between the WGS-based method and array-based methods on the same individuals. Fisher’s exact test was used to compare allele frequencies of these 67 variants between WGS and array genotype calls, as it offers greater accuracy when expected frequencies are low and does not assume a specific distribution. These analyses were performed on the entire cohort and further stratified by genetic ancestry and the HLA/non-HLA components of the GRS. We used R v4.1.1 on the UK Biobank RAP by DNAnexus for all statistical analyses.

## Results

### The T1DGRS derived from WGS is highly correlated to T1DGRS from array genotypes overall but lower in non-european genetic ancestries

**Fig. 1:**
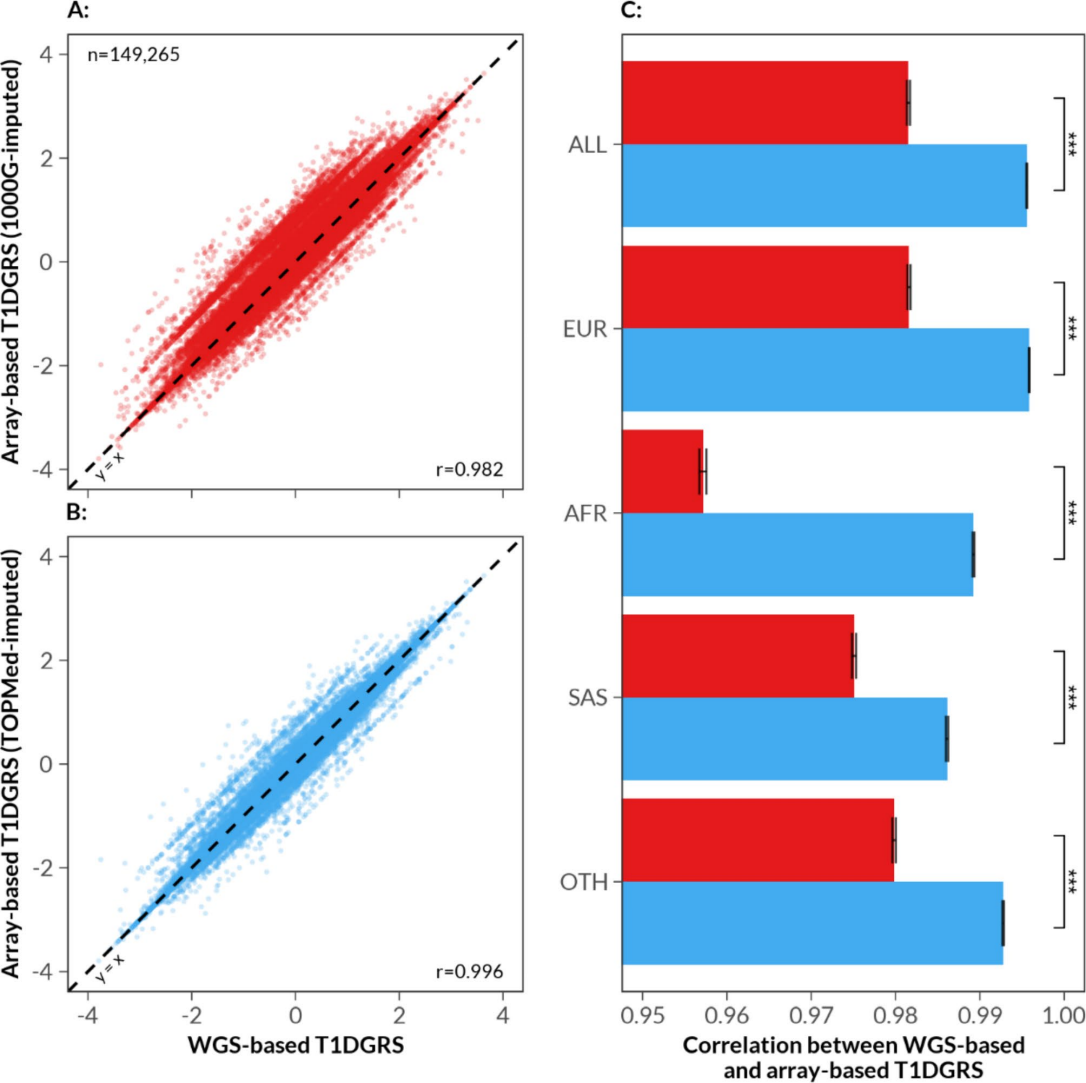
Correlation between T1DGRS derived from WGS versus derived from array genotypes imputed to the 1000 Genomes reference panel and the TOPMed reference panel. (**A**) Scatter plot of T1DGRS based on WGS against T1DGRS based on 1000 Genomes reference panel imputed array genotypes (in red) and (**B**) against T1DGRS based on TOPMed reference panel imputed array genotypes (in blue). (**C**) Bar chart showing Spearman’s rank correlation coefficient with 95% confidence intervals between WGS-based vs. 1000 Genomes-imputed array-based T1DGRS (red) and WGS-based vs. TOPMed-imputed array-based T1DGRS (blue), for all individuals and stratified by genetic ancestry (EUR/European, *n* = 137,888; AFR/African, *n* = 2,404; SAS/South Asian, *n* = 3,346; OTH/Others, *n* = 5,627). *** *p* < 0.001 from the correlation z-test (by Meng et al.).

The correlation between WGS-based T1DGRS and 1000 Genomes-imputed array-based T1DGRS was 0.9815 (95% CI 0.9813, 0.9817) (Fig. 1A). In contrast, WGS-based T1DGRS showed a higher, near-perfect correlation to TOPMed-imputed array-based T1DGRS (0.99555; 95% CI 0.99551, 0.99560) (Fig. 1B). The correlation was also higher, across all genetic ancestries, in the TOPMed-imputed array-based score than in the 1000 Genomes-imputed array-based score, particularly for the African and South Asian genetic ancestries (0.9892; 95% CI 0.9891, 0.9893 and 0.9861; 95% CI 0.9860, 0.9862 respectively) (Fig. 1C). The results were consistent for individuals with WGS-based T1DGRS ≥ 90^th^ centile, across both HLA and non-HLA components of the GRS (Suppl. Figure 1, Suppl. Figure 2).

### The differences in minor allele frequencies contribute to the discrepancies between WGS-based and array-based T1DGRS

**Fig. 2:**
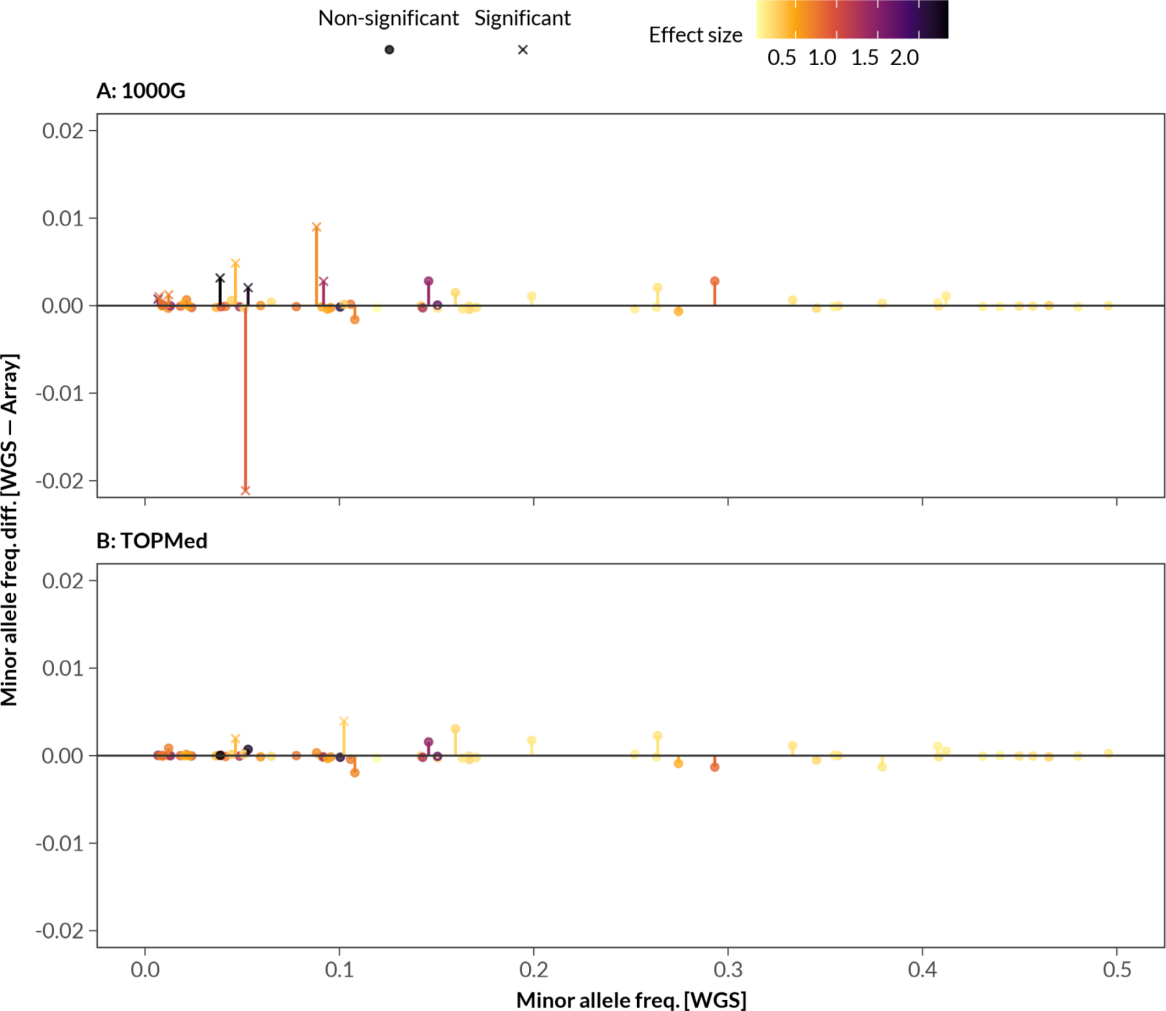
Minor allele frequency differences between WGS and imputed array genotypes. Bar graph showing minor allele frequency differences between WGS and (**A**) 1000 Genomes-imputed and (**B**) TOPMed-imputed genotypes for all individuals. Absolute effect sizes for each variant are also shown. Allele frequency association was performed between WGS-derived and array-derived variants using the Fisher’s exact test, considered significant when below a Bonferroni-corrected threshold p-value of 7.4 × 10^− 4^ (0.05/67).

The WGS-based T1DGRS values were on average 0.043 SD lower than their corresponding 1000 Genomes-imputed array-based T1DGRS values (95% CI -0.044, -0.042; *p* < 10^− 300^), and 0.0028 SD lower than corresponding TOPMed-imputed array-based T1DGRS values (95% CI -0.0033, -0.0023; *p* < 10^− 31^) (Suppl. Figure 3 A). Bland-Altman analysis on all individuals revealed no proportional bias between WGS-based T1DGRS and array-based T1DGRS (Suppl. Figure 4). The 1000 Genomes-imputed array-based T1DGRS was much lower across all ancestries whereas TOPMed-imputed array-based T1DGRS was much closer to WGS-based T1DGRS (mean difference range 0.003–0.019 SD) (Suppl. Figure 3 A). In line with these results, the minor allele frequencies for 9 variants were statistically different between the WGS and the 1000 Genomes-imputed array genotypes after Bonferroni correction, whereas only 2 variants were different for the TOPMed-imputed array genotypes (Fig. 2, Suppl. Table 3). Further investigation revealed that the differences were predominantly from the non-European genetic ancestries and the lower frequency variants in 1000 Genomes-imputed data (Suppl. Figure 5, Suppl. Table 3). These data together suggest that differences in minor allele frequencies contribute to the discrepancies between WGS-based and array-based T1DGRS.

### Array-based T1DGRS categorised up to 5% of cases differently at clinically relevant T1DGRS risk thresholds

We next assess the impact of different method on clinically utilised thresholds using WGS-based T1DGRS as a reference. Of the 74,633 individuals with WGS-based T1DGRS < 50^th^ centile (threshold for excluding T1D in clinical practice)^2,6^, 4.4% (95% CI 4.3%, 4.6%) would categorise to have T1DGRS ≥ 50^th^ centile using 1000 Genomes-imputed array-based T1DGRS and 1.1% (95% CI 1.0%, 1.2%) using TOPMed-imputed array-based T1DGRS (Table 1). Similarly, of the 14,924 individuals with WGS-based T1DGRS ≥ 90^th^ centile (threshold for T1D screening in the population)^2^, 4.2% (95% CI 3.8%, 4.5%) were found to have score below this threshold using 1000 Genomes-imputed array-based T1DGRS and 2.3% (95% CI 2.0%, 2.5%) using TOPMed-imputed array-based T1DGRS. Overall, the accuracy of 1000 Genomes-imputed array-based T1DGRS at the 50^th^ and 90^th^ centile thresholds were 97.2% and 98.3%, and were higher for TOPMed-imputed array-based T1DGRS (99% and 99.5% respectively). In line with this higher accuracy, the discriminative ability of the T1DGRS for European T1D cases (*n* = 112) and European controls was similar across all the methods (ROCAUC 0.93; 95% CI 0.9, 0.95 for all) (Suppl. Figure 6). We were unable to perform this analysis in non-European ancestries due to lack of T1D cases in our study cohort.

**Table 1:** Tables showing the accuracy of array-derived T1DGRS at clinically useful levels of disease risk, using WGS-derived T1DGRS as a reference.

		Array-based (1000G-imputed) T1DGRS centiles % (*N*)		Array-based (TOPMed-imputed) T1DGRS centiles % (*N*)	
		< 50th	≥ 50th	< 50th	≥ 50th
WGS-based T1DGRS centiles (N)	< 50^th^ (74,633)	95.6 (71,316)	4.4 (3,317)	98.9 (73,817)	1.1 (816)
	≥ 50^th^ (74,632)	1.07 (802)	98.93 (73,830)	0.86 (640)	99.14 (73,992)

		Array-based (1000G-imputed) T1DGRS centiles % (*N*)		Array-based (TOPMed-imputed) T1DGRS centiles % (*N*)	
		< 90th	≥ 90th	< 90th	≥ 90th
WGS-based T1DGRS centiles (N)	< 90^th^ (134,341)	98.6 (132,410)	1.4 (1,931)	99.7 (133,953)	0.3 (388)
	≥ 90^th^ (14,924)	4.2 (616)	95.8 (14,308)	2.3 (337)	97.7 (14,587)

### WGS-derived T1DGRS were lower for individuals of African and south Asian ancestries compared to European ancestry

**Fig. 3:**
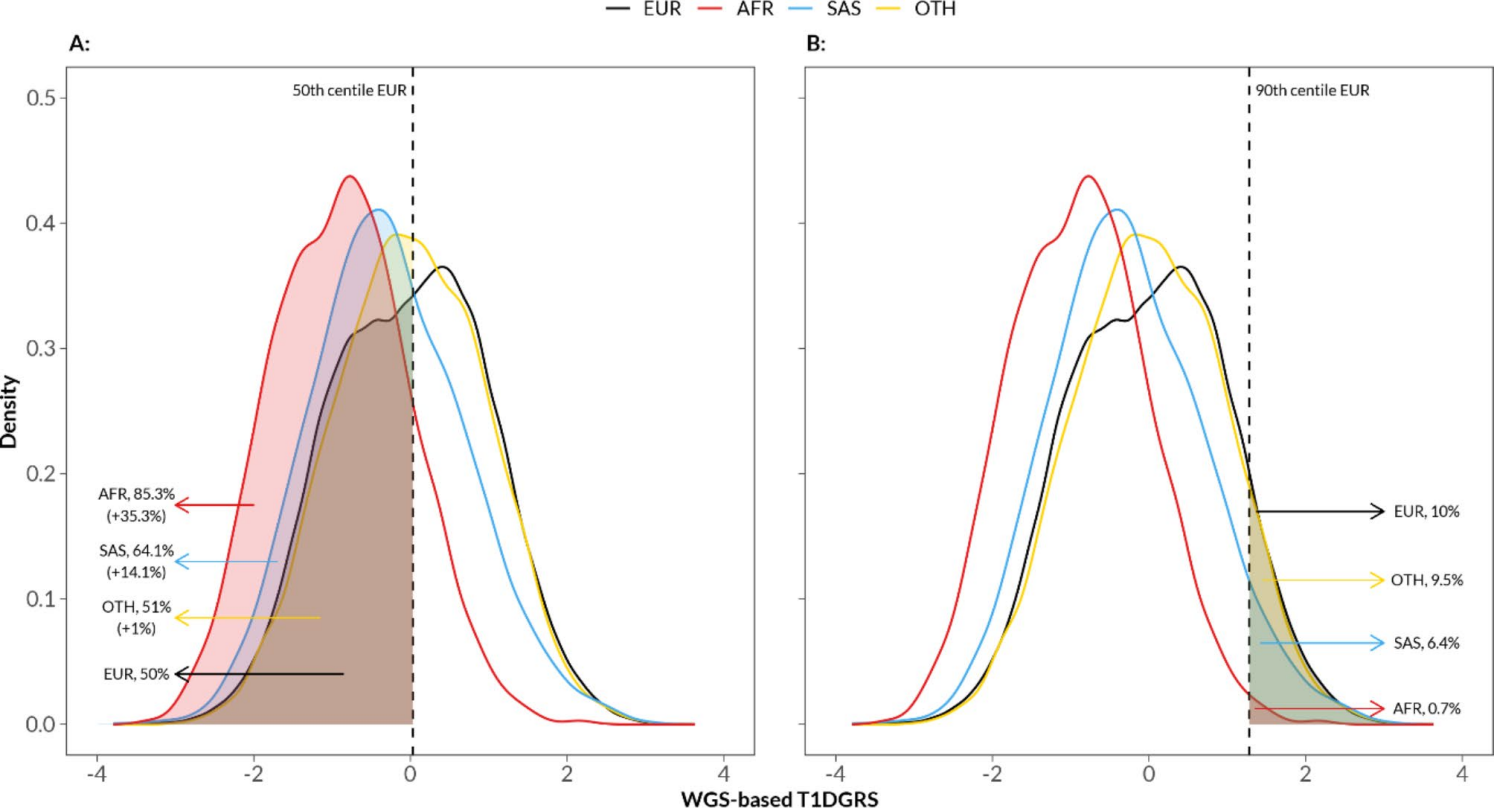
Density plot of standardised WGS-based T1DGRS stratified by genetic ancestry (EUR/European, *n* = 137,888; African/AFR, *n* = 2,404; SAS/South Asian, *n* = 3,346; OTH/Others, *n* = 5,627). Shows clinically relevant risk thresholds. (**A**) 50^th^ centile and (**B**) 90^th^ centile used in T1D screening processes, calculated based on the scores of the European genetic ancestry individuals, and the proportion of individuals captured by them.

Next-generation sequencing methods like WGS can effectively address the biases introduced by imputation reference panels in array-genotyping, facilitating better comparison of GRS across diverse ancestries. Currently, the T1DGRS threshold derived from European ancestry populations is proposed for use in clinical practice to classify diabetes subtypes and predict T1D. However, the performance of this European-centric threshold in other populations has not been explored. We therefore compare the T1DGRS across genetic ancestries using WGS-derived genotypes. We found that the WGS-based T1DGRS was substantially lower for individuals of African ancestry (-0.89 SD, 95% CI -0.92, -0.85; *p* < 10^− 300^) and South Asian ancestry (-0.28 SD, 95% CI -0.31, -0.24; *p* < 10^− 58^) compared to individuals of European ancestry (Fig. 3). This was found to correlate with the difference in allele frequency in non-Europeans compared to Europeans (Suppl. Figure 7, Suppl. Table 3). Use of the European ancestry-based population threshold of < 50^th^ centile (threshold for excluding T1D in clinical practice) will identify an additional 35.3% of African as having a low genetic risk of T1D (95% CI 33.8%, 36.7%; *p* < 10^− 290^) and an additional 14.1% South Asians (95% CI 12.4%, 15.7%; *p* < 10^− 60^). Similarly, use of the European ancestry-based population risk threshold of ≥ 90^th^ centile (threshold for T1D screening in the population) will only identify 0.71% (95% CI 0.41%, 1.13%; *p* < 10^− 300^) of individuals of African ancestry and 6.4% (95% CI 5.6%, 7.2%; *p* < 10^− 13^) of South Asian individuals rather than expected 10%. Results were consistent in both the HLA and non-HLA components of the score, with greater differences observed in non-HLA score across genetic ancestries (Suppl. Figure 8).

## Discussion

Our results advocate using TOPMed imputation as a reliable and cost-effective approach for generating T1DGRS in large studies. Array genotypes commonly require imputation using reference panels like 1000 Genomes or TOPMed before the analysis. Mean T1DGRS using the TOPMed imputation panel was approximately fifteen-fold closer to the WGS-based score compared to the 1000 Genomes-imputed array genotypes (0.0028 vs. 0.043 SD difference respectively). This improvement was more prominent for non-European genetic ancestries, particularly for the African ancestry individuals and reflected in clinically relevant thresholds showing a 2.5-fold lower re-categorisation with TOPMed-imputed versus 1000 Genomes-imputed genotypes (11.76% vs. 29.41%). This data aligns with previous studies in other disease or traits that also showed better imputation with the TOPMed reference panel in non-European populations^37^. The greater diversity in ancestry and larger sample size are likely underlying reasons for better performance of the TOPMed panel^27^.

The marginal gains from WGS do not appear to justify the substantially greater costs at present. The continual improvements to imputation reference panels such as TOPMed, will only further enhance array-based methods for polygenic scoring. For instance, the more recent TOPMed R3 reference panel, released in December 2023, includes 133,597 individuals – approximately 30% more sites and samples than TOPMed-r2, which was used by UK Biobank. This has improved the accuracy of imputation for non-European ancestries, particularly for individuals of African descent^10^. However, continent/ancestry-specific reference panels, despite being smaller, have been shown to perform equally well or better when they include diverse local populations^10^. The benefits of this strategy are likely to be more pronounced for non-European ancestries due to better linkage disequilibrium architecture and rarer ancestry specific variants. It’s important to note that while these advancements in reference panels are promising, their full impact on imputation accuracy and subsequent genetic analyses and clinical impact across diverse populations requires further investigation.

Caution is warranted regarding the indiscriminate use of European-centric T1DGRS risk thresholds in clinical applications. Such practices may lead to the exclusion of non-Europeans from T1D screening programmes and increase unnecessary monogenic diabetes testing. A T1DGRS below the 50^th^ centile of a European population (equivalent to the 5^th^ centile of T1D cases) can aid in selecting individuals for monogenic diabetes genetic testing in autoantibody-negative cases by excluding cases with antibody-negative T1D^4–6^. Despite using WGS data that overcomes previous imputation-related biases, we demonstrate that employing this threshold will result in more individuals being selected for expensive monogenic diabetes testing (an additional 35.3% of African ancestry individuals and 14.1% of South Asians). Conversely, applying the European ancestry-derived 90th centile risk threshold to select individuals for T1D screening would only capture 0.71% of African ancestry individuals and 6.4% of South Asians rather than the expected 10%, thus missing out on the potential benefits of these initiatives. These data strongly support recent calls for developing T1DGRS thresholds tailored to genetic ancestry, for enabling equitable precision screening and prevention across diverse populations^38^. However, the large genetic diversity within South Asian and African populations and admixture in population means that a single ancestry specific threshold may not be appropriate across all ancestral specific subgroups. Since ancestry exists on a continuum, defining distinct subgroups for separate standards is challenging and may limit applicability. One alternative is to generate risk scores with better transferability across ancestries using previously published methods^39^. However, large multi-ancestry GWAS studies in type 1 diabetes are needed to develop robust cross-ancestry scores. Until more data enables rigorous threshold derivation, careful consideration is needed when applying European-based risk thresholds to understudied groups.

Our study focused on the T1DGRS developed by Sharp et al. (referred to as T1DGRS2 in the original publication)^3^. Although this score is widely used, there are other versions and methods of calculating genetic risk for T1D that were not investigated in our current study. While genome-wide polygenic scores are gaining popularity, they may not be suitable for capturing the dominant HLA risk in T1D due to complex DR-DQ haplotype interactions, though they could potentially better capture the less contributory non-HLA components. We deliberately chose to not generate a new T1DGRS as our aim was to assess a previously validated score. Further research will be needed as new versions of the score become available, including those derived from large multi-ancestry GWAS or machine learning methods that better capture HLA interactions. However, while these new scoring approaches may improve performance across ancestries, they are unlikely to overcome the fundamental differences between WGS sequencing and array genotype methods.

The lower T1DGRS in the African and South Asian populations can be due to multiple reasons. The lower score could represent the use of association and variants from European-centric discovery studies (i.e. false low) or true difference due to the combination of genetic drift, greater genetic diversity, differing gene-environment interactions^40,41^. Improved capture of T1D risk in African individuals when the discovery dataset focused on the African genetic ancestry, supports that these differences could be due to biased data^42^. However, the lower and variable incidence of T1D in African and South Asian countries may suggest that lower population risk could be a true observation^43,44^. Further well-powered studies in non-European ancestry populations are needed to better understand the relative contributions of these factors to the observed disparities in T1DGRS and improve risk assessment in these groups.

We used whole genome sequencing as the standard for comparison. However, some genotypes, particularly in the HLA region, may have included false-positive calls. Our robust quality control filtering of all included variants indicates false positives are likely to be quite low. We did not perform imputation using ancestry-specific reference panels, which could potentially improve accuracy for underrepresented groups compared to standard multi-ancestry panels like 1000 Genomes or TOPMed. However, such imputations are not commonly used and thus were not a focus of our study. However, it will be useful to assess these in the future studies. Our study did not aim to assess the transferability of the risk loci to non-European ancestries but propose this would be a next logical step now that we have shown that difference is not due to imputation. We also lack sufficient data in the UK Biobank (< 10 T1D cases in these ancestries) to provide such data. However, the similar discriminative ability of the same GRS that we used in the current study to identify T1D cases across racial/ethnic groups in a youth cohort from the USA, suggests there is some transferability of loci^35^.

While this is the largest study of a T1DGRS from WGS in the population, our study cohort has inherent limitations. It exhibits a healthy volunteer bias and is predominantly of European ancestry, with a limited, albeit large, number of non-European ancestry participants. This was reflected in the lack of young T1D cases in non-European ancestries. We demonstrated that TOPMed imputation of array genotype data performs nearly as well as WGS. However, there are practical limitations to using these resources. Utilising TOPMed data may be challenging due to sample confidentiality agreements. One must upload their array genotypes to a remote server for imputation, which requires substantial computing resources and expertise. These factors present difficulties, particularly for low- and middle-income countries.

In conclusion, whole-genome sequencing emerges as a viable alternative for generating T1DGRS in large-scale studies. We advocate for the utilisation of TOPMed-imputed array genotypes as a cost-effective substitute. Our findings demonstrate that T1DGRS is lower in non-European ancestries at the population level, even when using WGS and European-centric T1DGRS thresholds in clinical practice can lead to lower diabetes classification and prediction of type 1 diabetes in non-European ancestries. We recommend future research focus on large multi-ancestry genetic studies in type 1 diabetes to develop robust cross-ancestry scores. This approach would improve clinical utility in diabetes classification and prediction of T1D.

## Electronic supplementary material

Below is the link to the electronic supplementary material.

Supplementary Material 1

## Data Availability

All the data used in this study is freely accessible from the UK Biobank https://www.ukbiobank.ac.uk. The open-access Python package t1dgrs2 used in this analysis has been published to the software distribution channel Bioconda, available at https://anaconda.org/bioconda/t1dgrs2. It uses quality-controlled genotype calls in standard PLINK v1.90 format along with 7 configuration files (containing the variants used and their corresponding effect alleles and weights), all downloadable from the “data” directory at https://github.com/t2diabetesgenes/t1dgrs2.
