# Supplementary material for: Type 1 diabetes genetic risk score variation across ancestries using whole genome sequencing and array-based approaches
Source: Sci Rep. 2024 Dec 28;14:31044. doi: 10.1038/s41598-024-82278-x (PMC11680773; doi:10.1038/s41598-024-82278-x)
Supplement: Supplementary file 1 — Supplementary Material 1 [file 41598_2024_82278_MOESM1_ESM.pdf]

Supplementary Table 1

Type 1 Diabetes associated variants used in this study for calculating the GRS (corresponding GRCh37 loci were used to score the 1000 Genomes-imputed array genotypes), along with their corresponding 1000 Genomes imputation INFO score & TOPMed imputation R^2:

	rsID	Locus (GRCh38)	Risk Allele	Weight	Type	INFO score	R^2
1	rs6679677	chr1-113761186-C-A	A	0.64	Non-HLA	1.000	0.991
2	rs3024505	chr1-206766559-G-A	A	-0.15	Non-HLA	1.000	1.003
3	rs2111485	chr2-162254026-A-G	G	0.16	Non-HLA	1.000	0.999
4	rs3087243	chr2-203874196-G-A	A	-0.17	Non-HLA	1.000	0.997
5	rs17388568	chr4-122408207-G-A	A	0.12	Non-HLA	1.000	0.994
6	rs9500974	chr6-29760476-G-T	T	0.63	HLA	0.992	0.993
7	rs1233320	chr6-29840255-C-G	G	0.74	HLA	1.000	0.996
8	rs72848653	chr6-29868825-C-T	T	0.78	HLA	0.997	1.000
9	rs9259118	chr6-29883132-T-C	T	0.31	HLA	0.999	1.000
10	rs12153924	chr6-29946312-G-A	A	0.44	HLA	0.997	1.000
11	rs12189871	chr6-31284147-C-T	T	0.45	HLA	1.000	0.997
12	rs540653847	chr6-31307016-G-GC	GC	1.78	HLA	0.963	0.989
13	rs9266268	chr6-31358273-G-A	A	0.39	HLA	1.000	1.000
14	rs16899379	chr6-31375490-G-A	A	-0.83	HLA	0.967	0.992
15	rs149663102	chr6-31376405-TG-T	T	-0.94	HLA	0.999	1.000
16	rs2524277	chr6-31439802-G-A	A	-0.60	HLA	1.000	1.000
17	rs9268500	chr6-32408740-C-T	C	1.24	HLA	1.000	1.000
18	rs17840116	chr6-32415221-G-C	C	-0.63	HLA-DQ69	0.998	0.992
19	rs75658393	chr6-32427740-T-C	T	0.81	HLA	0.996	0.991
20	rs9269173	chr6-32479411-T-A	A	0.67	HLA	0.900	0.983
21	rs9271347	chr6-32615766-A-G	G	1.69	HLA	0.999	1.000
22	rs1281935	chr6-32616043-G-T	T	-0.65	HLA-DQ73	0.901	0.993
23	rs1281943	chr6-32630313-T-C	C	0.90	HLA	0.919	0.991
24	rs9405117	chr6-32634974-C-A	A	1.39	HLA-DQ93	1.000	0.992
25	rs9469200	chr6-32635435-T-C	C	-1.32	HLA-DQ75	0.992	0.993
26	rs9273032	chr6-32644083-A-G	A	-1.43	HLA-DQ62	0.993	1.000
27	rs17211699	chr6-32658260-G-T	T	-0.75	HLA-DQ22	0.993	0.992
28	rs117806464	chr6-32658670-G-A	A	0.48	HLA-DQ61	1.000	0.994
29	rs9273369	chr6-32658707-T-C	C	1.26	HLA-DQ25	1.000	1.000
30	rs28746898	chr6-32680817-A-G	G	-2.35	HLA-DQ92	0.970	1.000
31	rs62406889	chr6-32704437-G-T	T	-2.22	HLA-DQ63	0.980	0.994
32	rs9275490	chr6-32705608-C-G	G	2.08	HLA-DQ81	1.000	0.997
33	rs1794265	chr6-32706960-C-G	G	-0.89	HLA-DQ53	0.999	0.996
34	rs10947332	chr6-32709663-G-A	A	-0.03	HLA-DQ51	1.000	1.000
35	rs12527228	chr6-32712215-C-T	T	-0.46	HLA-DQ42	0.987	0.987
36	rs6934289	chr6-33077179-T-C	C	-0.68	HLA	0.998	1.000
37	rs17214657	chr6-33079396-T-C	C	-0.19	HLA	0.998	0.996
38	rs2567287	chr6-33081408-G-A	A	0.84	HLA	0.997	0.993
39	rs9378176	chr6-33081532-A-G	G	-0.49	HLA	1.000	1.000
40	rs3129197	chr6-33103130-G-A	A	0.24	HLA	1.000	1.000
41	rs72928038	chr6-90267049-G-A	A	0.18	Non-HLA	1.000	1.000
42	rs9388489	chr6-126377573-A-G	A	0.16	Non-HLA	1.000	0.998
43	rs1738074	chr6-159044945-T-C	C	0.08	Non-HLA	1.000	0.992

44	rs4948088	chr7-50959497-A-C	C	0.26	Non-HLA	1.000	1.000
45	rs6476839	chr9-4290823-A-T	T	0.11	Non-HLA	0.998	1.000
46	rs61839660	chr10-6052734-C-T	T	-0.48	Non-HLA	1.000	0.994
47	rs41295121	chr10-6087680-C-T	T	-0.71	Non-HLA	0.955	0.959
48	rs60888743	chr10-88291560-A-G	A	0.18	Non-HLA	0.994	0.986
49	rs3842753	chr11-2159830-T-G	G	0.83	Non-HLA	0.988	0.970
50	rs10492166	chr12-9733403-G-A	A	-0.14	Non-HLA	0.996	0.992
51	rs11170466	chr12-53192075-C-T	T	0.17	Non-HLA	0.997	1.000
52	rs4759229	chr12-56080696-A-G	A	0.22	Non-HLA	0.997	0.991
53	rs653178	chr12-111569952-C-T	C	0.26	Non-HLA	1.000	1.000
54	rs9585056	chr13-99429512-C-T	C	0.11	Non-HLA	1.000	0.999
55	rs56994090	chr14-100840110-T-C	C	-0.13	Non-HLA	0.997	0.994
56	rs72727394	chr15-38554821-C-T	T	0.14	Non-HLA	0.991	0.986
57	rs2289702	chr15-78944951-C-T	C	0.28	Non-HLA	0.990	0.981
58	rs12708716	chr16-11086016-A-G	G	-0.19	Non-HLA	1.000	1.000
59	rs9924471	chr16-28580209-G-A	A	0.22	Non-HLA	0.992	0.980
60	rs1893217	chr18-12809341-A-G	G	0.19	Non-HLA	1.000	1.000
61	rs1615504	chr18-69859408-T-C	C	0.12	Non-HLA	0.999	0.992
62	rs144309607	chr19-10381598-C-T	T	-0.40	Non-HLA	0.951	0.982
63	rs425105	chr19-46705224-T-C	T	0.15	Non-HLA	1.000	0.993
64	rs2281808	chr20-1629905-T-C	C	0.10	Non-HLA	1.000	0.999
65	rs9981624	chr21-42405613-G-C	C	0.17	Non-HLA	0.996	0.992
66	rs5763779	chr22-30108663-A-G	A	0.15	Non-HLA	0.995	0.992
67	rs229541	chr22-37195278-G-A	A	0.10	Non-HLA	1.000	1.000

Supplementary Table 2

Characteristics of individuals with type 1 diabetes versus controls. Values are summarised as either mean (SD) for continuous data or N (%) for categorical data.

	Type 1 diabetes N=121	Non-diabetes controls N=149,144
Age at recruitment, yr	53.2 (7.9)	57.0 (8.1)
Female	62 (51.2%)	82,213 (56.3%)
BMI, kg/m^2	26.7 (3.9)	27.4 (4.7)
HbA1c, mmol/mol	63.8 (13.2)	38.3 (6.3)
Age at diagnosis, yr	11.9 (4.3)	–
Genetic ancestry		
• European	112 (92.6%)	127,423 (93.1%)
• African	–	1,951 (1.4%)
• South Asian	–	2,475 (1.8%)
• Others	9 (7.4%)	5,032 (3.7%)

Supplementary Table 3

Minor allele frequency association testing using Fisher's exact test adjusted for multiple testing with Bonferroni correction for all variants, for both all participants and stratified by genetic ancestry, with significant results highlighted in bold:

Genetic ancestry	Locus (GRCh38)	Allele frequency			Fisher's exact p-value (threshold = 7.64×10^{-4})	
		WGS	1000G	TOPMed	1000G	TOPMed
All	chr1-113761186-C-A	0.096	0.096	0.096	0.7614	0.8395
	chr1-206766559-G-A	0.151	0.151	0.151	0.7556	0.7556
	chr2-162254026-A-G	0.408	0.408	0.409	0.9706	0.9119
	chr2-203874196-G-A	0.450	0.450	0.450	0.9772	0.973
	chr4-122408207-G-A	0.263	0.263	0.263	0.8762	0.8994
	chr6-29760476-G-T	0.009	0.009	0.009	0.6138	0.9027
	chr6-29840255-C-G	0.041	0.042	0.042	0.8762	0.8456
	chr6-29868825-C-T	0.078	0.078	0.078	0.8772	1
	chr6-29883132-T-C	0.142	0.142	0.142	0.9734	0.9527
	chr6-29946312-G-A	0.275	0.275	0.275	0.5734	0.4369
	chr6-31284147-C-T	0.091	0.091	0.091	0.8537	0.8678
	chr6-31307016-G-GC	0.007	0.006	0.007	***3.27×10^{-4}**	0.8738
	chr6-31358273-G-A	0.037	0.037	0.037	0.6695	0.967
	chr6-31375490-G-A	0.007	0.006	0.007	***1.23×10^{-6}**	0.9755
	chr6-31376405-TG-T	0.039	0.039	0.039	0.8104	0.9521
	chr6-31439802-G-A	0.059	0.059	0.060	0.9913	0.8353
	chr6-32408740-C-T	0.049	0.049	0.049	0.8286	0.9233
	chr6-32415221-G-C	0.012	0.012	0.012	0.3035	0.9525
	chr6-32427740-T-C	0.018	0.018	0.018	0.8466	0.9923
	chr6-32479411-T-A	0.021	0.021	0.021	0.07393	0.7265
	chr6-32615766-A-G	0.151	0.150	0.151	0.9423	0.925
	chr6-32616043-G-T	0.088	0.079	0.088	***8.06×10^{-35}**	0.6541
	chr6-32630313-T-C	0.052	0.073	0.051	***1.54×10^{-245}**	0.5696
	chr6-32634974-C-A	0.013	0.013	0.013	0.9456	0.9818
	chr6-32635435-T-C	0.092	0.089	0.092	***1.86×10^{-4}**	0.8437
	chr6-32644083-A-G	0.146	0.143	0.144	0.00216	0.08833
	chr6-32658260-G-T	0.106	0.106	0.106	0.8462	0.599
	chr6-32658670-G-A	0.009	0.009	0.009	0.8784	0.8784
	chr6-32658707-T-C	0.143	0.143	0.143	0.7815	0.8302
	chr6-32680817-A-G	0.039	0.035	0.039	***1.08×10^{-10}**	0.9571
	chr6-32704437-G-T	0.053	0.051	0.052	***3.59×10^{-4}**	0.2168
	chr6-32705608-C-G	0.100	0.101	0.101	0.8464	0.8162
	chr6-32706960-C-G	0.024	0.024	0.024	0.5948	0.9259
	chr6-32709663-G-A	0.119	0.120	0.120	0.7616	0.7616
	chr6-32712215-C-T	0.020	0.020	0.020	0.4838	0.9927
	chr6-33077179-T-C	0.108	0.110	0.110	0.04988	0.01599
	chr6-33079396-T-C	0.065	0.065	0.065	0.5381	0.9122
	chr6-33081408-G-A	0.009	0.009	0.009	0.794	0.9562
	chr6-33081532-A-G	0.022	0.022	0.022	0.9374	0.993
	chr6-33103130-G-A	0.167	0.167	0.167	0.672	0.6544
	chr6-90267049-G-A	0.170	0.171	0.171	0.831	0.8203

	chr6-126377573-A-G	0.457	0.457	0.457	0.9648	0.973
	chr6-159044945-T-C	0.440	0.440	0.440	0.9376	0.9979
	chr7-50959497-A-C	0.045	0.044	0.044	0.2575	0.8115
	chr9-4290823-A-T	0.408	0.408	0.407	0.8083	0.4016
	chr10-6052734-C-T	0.094	0.094	0.094	0.601	0.6704
	chr10-6087680-C-T	0.012	0.011	0.011	***6.50×10^{-6}**	0.002301
	chr10-88291560-A-G	0.264	0.262	0.262	0.06951	0.04584
	chr11-2159830-T-G	0.293	0.290	0.295	0.01779	0.2692
	chr12-9733403-G-A	0.496	0.496	0.496	0.9917	0.8476
	chr12-53192075-C-T	0.051	0.051	0.051	0.6295	0.7999
	chr12-56080696-A-G	0.346	0.346	0.346	0.8146	0.6808
	chr12-111569952-C-T	0.465	0.465	0.465	0.9917	0.9111
	chr13-99429512-C-T	0.252	0.252	0.252	0.743	0.9003
	chr14-100840110-T-C	0.412	0.411	0.412	0.389	0.6889
	chr15-38554821-C-T	0.199	0.198	0.197	0.2976	0.09301
	chr15-78944951-C-T	0.102	0.102	0.098	0.8574	***4.54×10^{-7}**
	chr16-11086016-A-G	0.357	0.357	0.357	0.9741	1
	chr16-28580209-G-A	0.160	0.158	0.157	0.1177	0.001258
	chr18-12809341-A-G	0.167	0.167	0.167	0.9695	0.9585
	chr18-69859408-T-C	0.480	0.480	0.480	0.9215	0.9731
	chr19-10381598-C-T	0.047	0.042	0.045	***8.43×10^{-20}**	***3.01×10^{-4}**
	chr19-46705224-T-C	0.163	0.164	0.164	0.7106	0.7634
	chr20-1629905-T-C	0.355	0.355	0.355	0.9353	0.9676
	chr21-42405613-G-C	0.333	0.333	0.332	0.5992	0.345
	chr22-30108663-A-G	0.379	0.379	0.381	0.8264	0.3112
	chr22-37195278-G-A	0.431	0.431	0.431	0.9417	0.9646
European	chr1-113761186-C-A	0.101	0.101	0.101	0.7715	0.8476
	chr1-206766559-G-A	0.154	0.154	0.154	0.7683	0.7683
	chr2-162254026-A-G	0.392	0.392	0.392	0.9626	0.8946
	chr2-203874196-G-A	0.451	0.452	0.452	0.9763	0.9827
	chr4-122408207-G-A	0.273	0.273	0.273	0.8823	0.9038
	chr6-29760476-G-T	0.008	0.007	0.008	0.6184	0.8769
	chr6-29840255-C-G	0.043	0.043	0.043	0.8836	0.8627
	chr6-29868825-C-T	0.076	0.076	0.076	0.8588	0.9959
	chr6-29883132-T-C	0.147	0.147	0.147	0.9818	0.9575
	chr6-29946312-G-A	0.283	0.284	0.284	0.6365	0.4749
	chr6-31284147-C-T	0.091	0.091	0.091	0.8476	0.8697
	chr6-31307016-G-GC	0.007	0.006	0.007	***4.62×10^{-4}**	0.9228
	chr6-31358273-G-A	0.037	0.037	0.037	0.7262	0.9658
	chr6-31375490-G-A	0.006	0.006	0.006	***2.41×10^{-5}**	1
	chr6-31376405-TG-T	0.040	0.040	0.040	0.9288	0.9561
	chr6-31439802-G-A	0.060	0.060	0.060	0.9683	0.8115
	chr6-32408740-C-T	0.051	0.051	0.051	0.8398	0.9365
	chr6-32415221-G-C	0.010	0.011	0.010	0.8222	0.9578
	chr6-32427740-T-C	0.019	0.019	0.019	0.9682	0.9841
	chr6-32479411-T-A	0.018	0.017	0.018	0.04862	0.8872
	chr6-32615766-A-G	0.150	0.150	0.150	0.9368	0.9278
	chr6-32616043-G-T	0.094	0.084	0.093	***2.14×10^{-34}**	0.6669
	chr6-32630313-T-C	0.055	0.077	0.055	***2.63×10^{-236}**	0.5823
	chr6-32634974-C-A	0.013	0.013	0.013	0.9524	0.9905

	chr6-32635435-T-C	0.089	0.086	0.089	***4.63×10^{-4}**	0.8055
	chr6-32644083-A-G	0.148	0.146	0.147	0.006269	0.1153
	chr6-32658260-G-T	0.107	0.106	0.107	0.8202	0.6038
	chr6-32658670-G-A	0.004	0.004	0.004	0.9355	0.9355
	chr6-32658707-T-C	0.147	0.147	0.147	0.7871	0.8372
	chr6-32680817-A-G	0.039	0.036	0.039	***3.77×10^{-10}**	0.9614
	chr6-32704437-G-T	0.053	0.051	0.052	***2.36×10^{-4}**	0.2673
	chr6-32705608-C-G	0.102	0.103	0.103	0.852	0.8139
	chr6-32706960-C-G	0.022	0.023	0.022	0.8773	0.9638
	chr6-32709663-G-A	0.120	0.120	0.120	0.7651	0.7619
	chr6-32712215-C-T	0.020	0.019	0.020	0.6185	0.9767
	chr6-33077179-T-C	0.108	0.110	0.110	0.04825	0.01834
	chr6-33079396-T-C	0.060	0.060	0.060	0.9184	0.8869
	chr6-33081408-G-A	0.008	0.008	0.008	0.841	0.9386
	chr6-33081532-A-G	0.021	0.021	0.021	0.9254	1
	chr6-33103130-G-A	0.169	0.170	0.170	0.685	0.6666
	chr6-90267049-G-A	0.178	0.179	0.179	0.8411	0.8549
	chr6-126377573-A-G	0.453	0.453	0.453	0.9633	0.9655
	chr6-159044945-T-C	0.435	0.435	0.435	0.9393	0.9805
	chr7-50959497-A-C	0.045	0.045	0.045	0.2456	0.8058
	chr9-4290823-A-T	0.402	0.402	0.401	0.8067	0.5938
	chr10-6052734-C-T	0.098	0.098	0.098	0.6214	0.6867
	chr10-6087680-C-T	0.011	0.010	0.010	0.001107	0.2479
	chr10-88291560-A-G	0.262	0.260	0.260	0.1299	0.2261
	chr11-2159830-T-G	0.288	0.287	0.289	0.1583	0.4804
	chr12-9733403-G-A	0.494	0.494	0.494	0.9162	0.9076
	chr12-53192075-C-T	0.051	0.051	0.051	0.5851	0.8876
	chr12-56080696-A-G	0.349	0.349	0.350	0.8254	0.7069
	chr12-111569952-C-T	0.485	0.485	0.485	0.9742	0.9248
	chr13-99429512-C-T	0.247	0.248	0.247	0.7502	0.9651
	chr14-100840110-T-C	0.415	0.414	0.415	0.4146	0.7928
	chr15-38554821-C-T	0.202	0.202	0.201	0.4677	0.2912
	chr15-78944951-C-T	0.104	0.103	0.101	0.5862	***2.34×10^{-4}**
	chr16-11086016-A-G	0.354	0.354	0.354	0.9753	0.9775
	chr16-28580209-G-A	0.159	0.158	0.157	0.1617	0.004233
	chr18-12809341-A-G	0.169	0.170	0.170	0.9656	0.9456
	chr18-69859408-T-C	0.475	0.475	0.474	0.9355	0.9892
	chr19-10381598-C-T	0.048	0.044	0.047	***3.30×10^{-18}**	8.85×10^{-4}
	chr19-46705224-T-C	0.164	0.165	0.165	0.7137	0.7602
	chr20-1629905-T-C	0.365	0.365	0.365	0.9398	0.9688
	chr21-42405613-G-C	0.338	0.337	0.337	0.5781	0.4581
	chr22-30108663-A-G	0.396	0.395	0.397	0.6427	0.57
	chr22-37195278-G-A	0.423	0.423	0.423	0.9261	0.95
African	chr1-113761186-C-A	0.005	0.005	0.005	1	1
	chr1-206766559-G-A	0.030	0.030	0.029	1	1
	chr2-162254026-A-G	0.167	0.167	0.167	1	0.9782
	chr2-203874196-G-A	0.190	0.190	0.189	1	0.9377
	chr4-122408207-G-A	0.022	0.022	0.022	1	1
	chr6-29760476-G-T	0.065	0.064	0.065	0.8678	1
	chr6-29840255-C-G	0.026	0.026	0.026	0.9493	1

chr6-29868825-C-T	0.012	0.012	0.012	0.9263	1
chr6-29883132-T-C	0.072	0.072	0.072	0.9685	1
chr6-29946312-G-A	0.094	0.096	0.096	0.7275	0.7539
chr6-31284147-C-T	0.066	0.066	0.067	1	0.9673
chr6-31307016-G-GC	0.001	0.001	0.001	1	1
chr6-31358273-G-A	0.030	0.031	0.030	0.9056	1
chr6-31375490-G-A	0.050	0.041	0.049	0.04324	0.9624
chr6-31376405-TG-T	0.003	0.005	0.003	0.1067	1
chr6-31439802-G-A	0.072	0.071	0.071	0.9056	0.8743
chr6-32408740-C-T	0.004	0.004	0.004	1	1
chr6-32415221-G-C	0.082	0.093	0.081	0.06564	0.8815
chr6-32427740-T-C	0.001	0.001	0.001	1	1
chr6-32479411-T-A	0.040	0.040	0.038	0.8754	0.673
chr6-32615766-A-G	0.169	0.169	0.169	1	0.9783
chr6-32616043-G-T	0.007	0.004	0.006	0.06616	0.7975
chr6-32630313-T-C	0.002	0.004	0.002	0.07249	1
chr6-32634974-C-A	0.001	0.001	0.001	1	1
chr6-32635435-T-C	0.115	0.104	0.114	0.1011	0.8476
chr6-32644083-A-G	0.218	0.215	0.218	0.7839	0.9802
chr6-32658260-G-T	0.102	0.105	0.103	0.6589	0.8927
chr6-32658670-G-A	0.001	0.001	0.001	1	1
chr6-32658707-T-C	0.062	0.062	0.062	1	1
chr6-32680817-A-G	0.008	0.008	0.008	1	1
chr6-32704437-G-T	0.021	0.026	0.021	0.1575	0.9436
chr6-32705608-C-G	0.032	0.032	0.032	1	1
chr6-32706960-C-G	0.009	0.016	0.009	9.95×10^{-4}	1
chr6-32709663-G-A	0.153	0.153	0.153	1	1
chr6-32712215-C-T	0.080	0.077	0.079	0.5678	0.9399
chr6-33077179-T-C	0.121	0.122	0.123	0.8753	0.8022
chr6-33079396-T-C	0.377	0.377	0.377	1	1
chr6-33081408-G-A	0.064	0.063	0.064	0.8346	1
chr6-33081532-A-G	0.003	0.003	0.003	1	1
chr6-33103130-G-A	0.057	0.057	0.057	1	1
chr6-90267049-G-A	0.010	0.010	0.010	1	1
chr6-126377573-A-G	0.161	0.162	0.161	1	1
chr6-159044945-T-C	0.298	0.299	0.298	0.9644	1
chr7-50959497-A-C	0.043	0.043	0.043	0.9598	0.9199
chr9-4290823-A-T	0.468	0.459	0.472	0.3757	0.758
chr10-6052734-C-T	0.004	0.004	0.004	1	1
chr10-6087680-C-T	0.000	0.000	0.000	1	1
chr10-88291560-A-G	0.359	0.354	0.349	0.6172	0.3276
chr11-2159830-T-G	0.194	0.186	0.186	0.3467	0.3161
chr12-9733403-G-A	0.488	0.490	0.487	0.9009	0.9178
chr12-53192075-C-T	0.037	0.036	0.033	0.8697	0.4042
chr12-56080696-A-G	0.359	0.364	0.361	0.6632	0.8625
chr12-111569952-C-T	0.028	0.028	0.028	1	1
chr13-99429512-C-T	0.436	0.437	0.436	0.9672	0.9671
chr14-100840110-T-C	0.427	0.428	0.423	0.934	0.6936
chr15-38554821-C-T	0.062	0.055	0.059	0.17	0.5184
chr15-78944951-C-T	0.016	0.033	0.013	***2.78×10^{-8}**	0.3003

	chr16-11086016-A-G	0.443	0.443	0.443	1	0.9836
	chr16-28580209-G-A	0.160	0.155	0.156	0.5298	0.6323
	chr18-12809341-A-G	0.046	0.046	0.046	1	1
	chr18-69859408-T-C	0.271	0.271	0.270	0.9817	0.945
	chr19-10381598-C-T	0.003	0.003	0.003	0.8504	1
	chr19-46705224-T-C	0.135	0.135	0.135	1	0.9762
	chr20-1629905-T-C	0.219	0.219	0.218	1	0.8821
	chr21-42405613-G-C	0.171	0.154	0.159	0.02438	0.1244
	chr22-30108663-A-G	0.025	0.029	0.025	0.2269	0.844
	chr22-37195278-G-A	0.338	0.339	0.337	0.9142	0.9655
South Asian	chr1-113761186-C-A	0.019	0.019	0.019	0.9497	1
	chr1-206766559-G-A	0.124	0.124	0.124	0.9372	0.8956
	chr2-162254026-A-G	0.455	0.455	0.455	0.9723	1
	chr2-203874196-G-A	0.404	0.404	0.404	1	1
	chr4-122408207-G-A	0.150	0.150	0.150	0.9807	1
	chr6-29760476-G-T	0.014	0.014	0.014	1	1
	chr6-29840255-C-G	0.013	0.013	0.013	1	1
	chr6-29868825-C-T	0.139	0.139	0.139	1	1
	chr6-29883132-T-C	0.071	0.072	0.072	0.9732	0.9732
	chr6-29946312-G-A	0.138	0.139	0.138	0.9202	0.98
	chr6-31284147-C-T	0.129	0.129	0.129	1	1
	chr6-31307016-G-GC	0.001	0.001	0.001	0.6042	0.8019
	chr6-31358273-G-A	0.026	0.027	0.026	0.8716	1
	chr6-31375490-G-A	0.002	0.002	0.002	0.6898	1
	chr6-31376405-TG-T	0.056	0.056	0.056	1	1
	chr6-31439802-G-A	0.065	0.065	0.065	0.972	1
	chr6-32408740-C-T	0.024	0.024	0.024	1	0.9099
	chr6-32415221-G-C	0.014	0.014	0.014	1	1
	chr6-32427740-T-C	0.010	0.013	0.010	0.1476	1
	chr6-32479411-T-A	0.115	0.117	0.113	0.7431	0.7232
	chr6-32615766-A-G	0.206	0.206	0.206	1	1
	chr6-32616043-G-T	0.006	0.007	0.006	0.7458	0.9138
	chr6-32630313-T-C	0.001	0.006	0.001	***4.63×10^{-7}**	1
	chr6-32634974-C-A	0.008	0.009	0.008	1	1
	chr6-32635435-T-C	0.102	0.102	0.103	0.9772	0.9773
	chr6-32644083-A-G	0.079	0.067	0.073	0.008365	0.2372
	chr6-32658260-G-T	0.100	0.099	0.100	0.84	1
	chr6-32658670-G-A	0.143	0.144	0.144	0.9803	0.9803
	chr6-32658707-T-C	0.120	0.120	0.120	0.9788	0.9788
	chr6-32680817-A-G	0.054	0.051	0.055	0.3514	0.9696
	chr6-32704437-G-T	0.077	0.074	0.075	0.4316	0.6015
	chr6-32705608-C-G	0.072	0.071	0.072	1	1
	chr6-32706960-C-G	0.086	0.084	0.086	0.8041	0.9507
	chr6-32709663-G-A	0.091	0.091	0.091	0.976	0.976
	chr6-32712215-C-T	0.010	0.010	0.010	1	1
	chr6-33077179-T-C	0.073	0.073	0.074	1	0.8417
	chr6-33079396-T-C	0.073	0.056	0.073	***4.45×10^{-5}**	0.9735
	chr6-33081408-G-A	0.010	0.010	0.010	1	1
	chr6-33081532-A-G	0.012	0.012	0.012	1	1
	chr6-33103130-G-A	0.184	0.185	0.185	0.9822	0.9822

	chr6-90267049-G-A	0.064	0.064	0.065	1	0.9157
	chr6-126377573-A-G	0.349	0.349	0.349	1	1
	chr6-159044945-T-C	0.394	0.395	0.396	0.9576	0.8733
	chr7-50959497-A-C	0.043	0.042	0.042	0.8305	0.864
	chr9-4290823-A-T	0.471	0.469	0.464	0.8486	0.433
	chr10-6052734-C-T	0.045	0.046	0.045	0.9008	0.9337
	chr10-6087680-C-T	0.078	0.067	0.061	0.01375	***1.46×10^{-4}**
	chr10-88291560-A-G	0.326	0.323	0.308	0.7093	0.03118
	chr11-2159830-T-G	0.179	0.173	0.186	0.3582	0.3642
	chr12-9733403-G-A	0.470	0.464	0.471	0.5065	0.9578
	chr12-53192075-C-T	0.029	0.028	0.028	0.8757	0.917
	chr12-56080696-A-G	0.237	0.237	0.238	1	0.8705
	chr12-111569952-C-T	0.116	0.116	0.116	1	1
	chr13-99429512-C-T	0.340	0.341	0.338	0.9709	0.7697
	chr14-100840110-T-C	0.302	0.300	0.299	0.7337	0.6632
	chr15-38554821-C-T	0.164	0.153	0.144	0.08493	0.001296
	chr15-78944951-C-T	0.133	0.128	0.097	0.3527	***1.59×10^{-10}**
	chr16-11086016-A-G	0.380	0.380	0.379	1	0.9574
	chr16-28580209-G-A	0.127	0.125	0.115	0.7742	0.02809
	chr18-12809341-A-G	0.185	0.185	0.185	1	1
	chr18-69859408-T-C	0.493	0.493	0.491	0.9586	0.8353
	chr19-10381598-C-T	0.029	0.021	0.021	0.002139	0.00318
	chr19-46705224-T-C	0.158	0.158	0.158	1	1
	chr20-1629905-T-C	0.166	0.166	0.166	0.9629	1
	chr21-42405613-G-C	0.336	0.336	0.330	1	0.5058
	chr22-30108663-A-G	0.116	0.113	0.116	0.5481	0.9345
	chr22-37195278-G-A	0.463	0.463	0.462	0.9862	0.9862
Others	chr1-113761186-C-A	0.047	0.047	0.047	0.9748	1
	chr1-206766559-G-A	0.129	0.129	0.129	0.9683	0.9841
	chr2-162254026-A-G	0.458	0.458	0.458	1	0.9466
	chr2-203874196-G-A	0.433	0.433	0.434	1	0.9571
	chr4-122408207-G-A	0.191	0.191	0.191	0.9865	0.9729
	chr6-29760476-G-T	0.020	0.020	0.020	1	1
	chr6-29840255-C-G	0.033	0.033	0.033	0.9403	0.9403
	chr6-29868825-C-T	0.116	0.115	0.115	0.9667	1
	chr6-29883132-T-C	0.098	0.099	0.099	0.9821	0.9822
	chr6-29946312-G-A	0.223	0.224	0.225	0.7852	0.7732
	chr6-31284147-C-T	0.087	0.087	0.087	1	1
	chr6-31307016-G-GC	0.005	0.004	0.005	0.5475	0.922
	chr6-31358273-G-A	0.041	0.042	0.041	0.8673	1
	chr6-31375490-G-A	0.009	0.008	0.009	0.3116	1
	chr6-31376405-TG-T	0.025	0.026	0.025	0.6127	1
	chr6-31439802-G-A	0.044	0.043	0.044	0.8958	1
	chr6-32408740-C-T	0.027	0.027	0.027	0.9671	1
	chr6-32415221-G-C	0.018	0.020	0.018	0.3516	0.8797
	chr6-32427740-T-C	0.020	0.020	0.020	0.7761	1
	chr6-32479411-T-A	0.039	0.039	0.039	0.9447	0.9726
	chr6-32615766-A-G	0.127	0.127	0.127	1	1
	chr6-32616043-G-T	0.035	0.035	0.035	0.7959	0.9712
	chr6-32630313-T-C	0.018	0.032	0.018	***2.88×10^{-11}**	1

	chr6-32634974-C-A	0.026	0.026	0.026	1	1
	chr6-32635435-T-C	0.154	0.150	0.153	0.4679	0.9705
	chr6-32644083-A-G	0.101	0.096	0.099	0.2415	0.5921
	chr6-32658260-G-T	0.090	0.090	0.090	0.9814	0.9814
	chr6-32658670-G-A	0.037	0.037	0.037	0.8878	0.8878
	chr6-32658707-T-C	0.094	0.095	0.095	0.9818	1
	chr6-32680817-A-G	0.021	0.019	0.021	0.1515	0.9261
	chr6-32704437-G-T	0.051	0.050	0.050	0.9272	0.7602
	chr6-32705608-C-G	0.101	0.101	0.101	0.9647	0.9823
	chr6-32706960-C-G	0.031	0.033	0.032	0.5202	0.8786
	chr6-32709663-G-A	0.117	0.117	0.117	0.9834	1
	chr6-32712215-C-T	0.020	0.020	0.020	0.7739	1
	chr6-33077179-T-C	0.112	0.112	0.113	0.8819	0.7508
	chr6-33079396-T-C	0.059	0.058	0.059	0.6702	0.9775
	chr6-33081408-G-A	0.016	0.016	0.016	1	1
	chr6-33081532-A-G	0.070	0.070	0.070	0.9792	1
	chr6-33103130-G-A	0.144	0.144	0.144	0.9093	0.9093
	chr6-90267049-G-A	0.107	0.107	0.107	0.9656	0.9656
	chr6-126377573-A-G	0.439	0.439	0.439	1	0.9893
	chr6-159044945-T-C	0.470	0.470	0.471	1	0.9574
	chr7-50959497-A-C	0.025	0.025	0.026	0.9662	0.9324
	chr9-4290823-A-T	0.459	0.457	0.455	0.8616	0.5912
	chr10-6052734-C-T	0.068	0.068	0.068	0.9157	0.9367
	chr10-6087680-C-T	0.010	0.007	0.008	0.0125	0.02348
	chr10-88291560-A-G	0.241	0.237	0.234	0.5506	0.2826
	chr11-2159830-T-G	0.264	0.254	0.269	0.0934	0.3649
	chr12-9733403-G-A	0.481	0.480	0.482	0.9359	0.9571
	chr12-53192075-C-T	0.077	0.077	0.077	1	0.9601
	chr12-56080696-A-G	0.319	0.318	0.319	0.8405	0.9542
	chr12-111569952-C-T	0.367	0.367	0.367	0.9779	0.9669
	chr13-99429512-C-T	0.238	0.239	0.239	0.9626	0.9875
	chr14-100840110-T-C	0.395	0.393	0.393	0.7842	0.7844
	chr15-38554821-C-T	0.196	0.191	0.189	0.3314	0.1512
	chr15-78944951-C-T	0.086	0.087	0.077	0.8121	0.01768
	chr16-11086016-A-G	0.363	0.363	0.362	0.9889	0.9557
	chr16-28580209-G-A	0.187	0.185	0.183	0.6671	0.5015
	chr18-12809341-A-G	0.140	0.140	0.140	1	0.954
	chr18-69859408-T-C	0.494	0.494	0.495	0.9044	0.8832
	chr19-10381598-C-T	0.029	0.025	0.028	0.1156	0.7776
	chr19-46705224-T-C	0.154	0.154	0.154	0.9411	0.9558
	chr20-1629905-T-C	0.260	0.260	0.260	0.9879	0.9879
	chr21-42405613-G-C	0.294	0.292	0.289	0.8368	0.4791
	chr22-30108663-A-G	0.285	0.289	0.289	0.5327	0.4575
	chr22-37195278-G-A	0.488	0.488	0.488	0.9894	1

Supplementary Table 4

Accuracy of array genotype-derived T1DGRS at clinically useful levels of disease risk, using WGS-derived T1DGRS as a reference and split by genetic ancestry. For example, using the WGS-derived 50th centile threshold, of the 67,677 Europeans having a T1DGRS classified to be below this threshold, 4.48% of them are misclassified to be above it when using the 1000 Genomes-imputed array-based T1DGRS.

			Array-based (1000 Genomes-imputed) T1DGRS centiles % (N)		Array-based (TOPMed-imputed) T1DGRS centiles % (N)	
			<50th	≥50th	<50th	≥50th
WGS-based T1DGRS centiles (N)	European	<50th (67,677)	95.52 (64,647)	4.48 (3,030)	98.97 (66,981)	1.03 (696)
		≥50th (70,211)	0.96 (673)	99.04 (69,538)	0.78 (547)	99.22 (69,664)
	African	<50th (2,031)	97.05 (1,971)	2.95 (60)	98.72 (2,005)	1.28 (26)
		≥50th (373)	10.19 (38)	89.81 (335)	4.56 (17)	95.44 (356)
	South Asian	<50th (2,113)	95.84 (2,025)	4.16 (88)	98.01 (2,071)	1.99 (42)
		≥50th (1,233)	3.16 (39)	96.84 (1,194)	2.92 (36)	97.08 (1,197)
	Others	<50th (2,812)	95.06 (2,673)	4.94 (139)	98.15 (2,760)	1.85 (52)
		≥50th (2,815)	1.85 (52)	98.15 (2,763)	1.42 (40)	98.58 (2,775)
			<90th	≥90th	<90th	≥90th
WGS-based T1DGRS centiles (N)	European	<90th (123,744)	98.51 (121,896)	1.49 (1,848)	99.71 (123,387)	0.29 (357)
		≥90th (14,144)	3.98 (563)	96.02 (13,581)	2.10 (297)	97.90 (13,847)
	African	<90th (2,387)	99.92 (2,385)	0.08 (2)	99.96 (2,386)	0.04 (1)
		≥90th (17)	29.41 (5)	70.59 (12)	11.76 (2)	88.24 (15)
	South Asian	<90th (3,126)	99.46 (3,109)	0.54 (17)	99.65 (3,115)	0.35 (11)
		≥90th (220)	6.36 (14)	93.64 (206)	7.27 (16)	92.73 (204)
	Others	<90th (5,084)	98.74 (5,020)	1.26 (64)	99.63 (5,065)	0.37 (19)
		≥90th (543)	6.26 (34)	93.74 (509)	4.05 (22)	95.95 (521)

Supplementary Figure 1

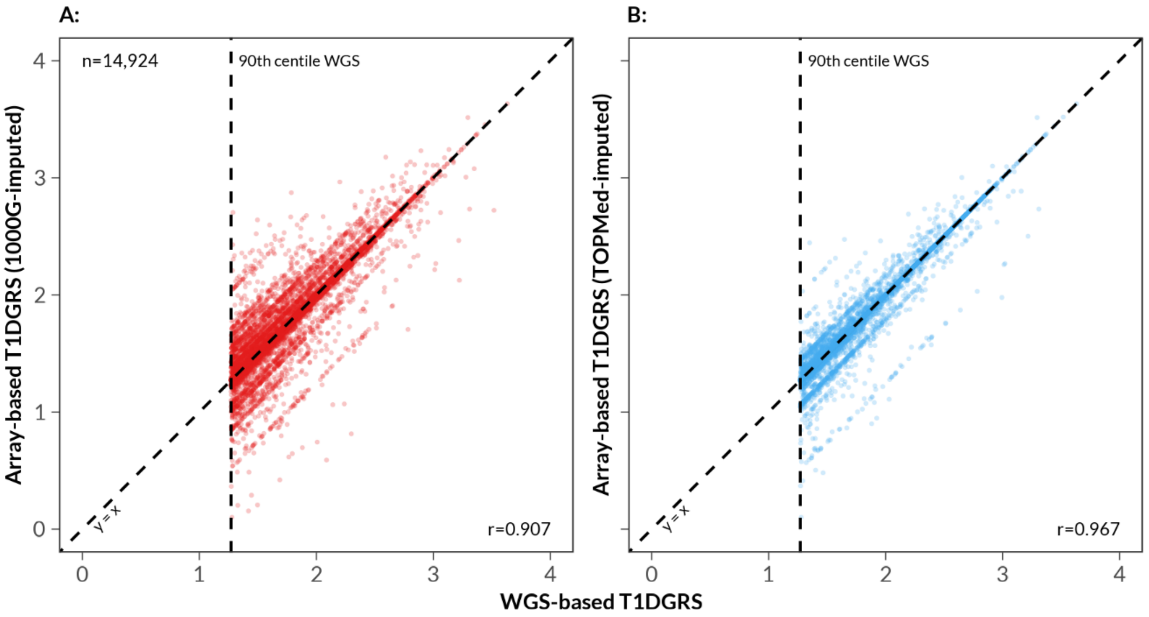

Supplementary Figure 1: Scatter plot and Spearman correlation between T1DGRS derived from the 90th centile and above from WGS versus corresponding scores from array genotypes*, imputed to* ***(A)*** *the 1000 Genomes reference panel and* ***(B)*** *the TOPMed panel, showing reduced correlation compared to the correlation using all individuals, with a more pronounced effect with the 1000 Genomes panel.*

Supplementary Figure 2

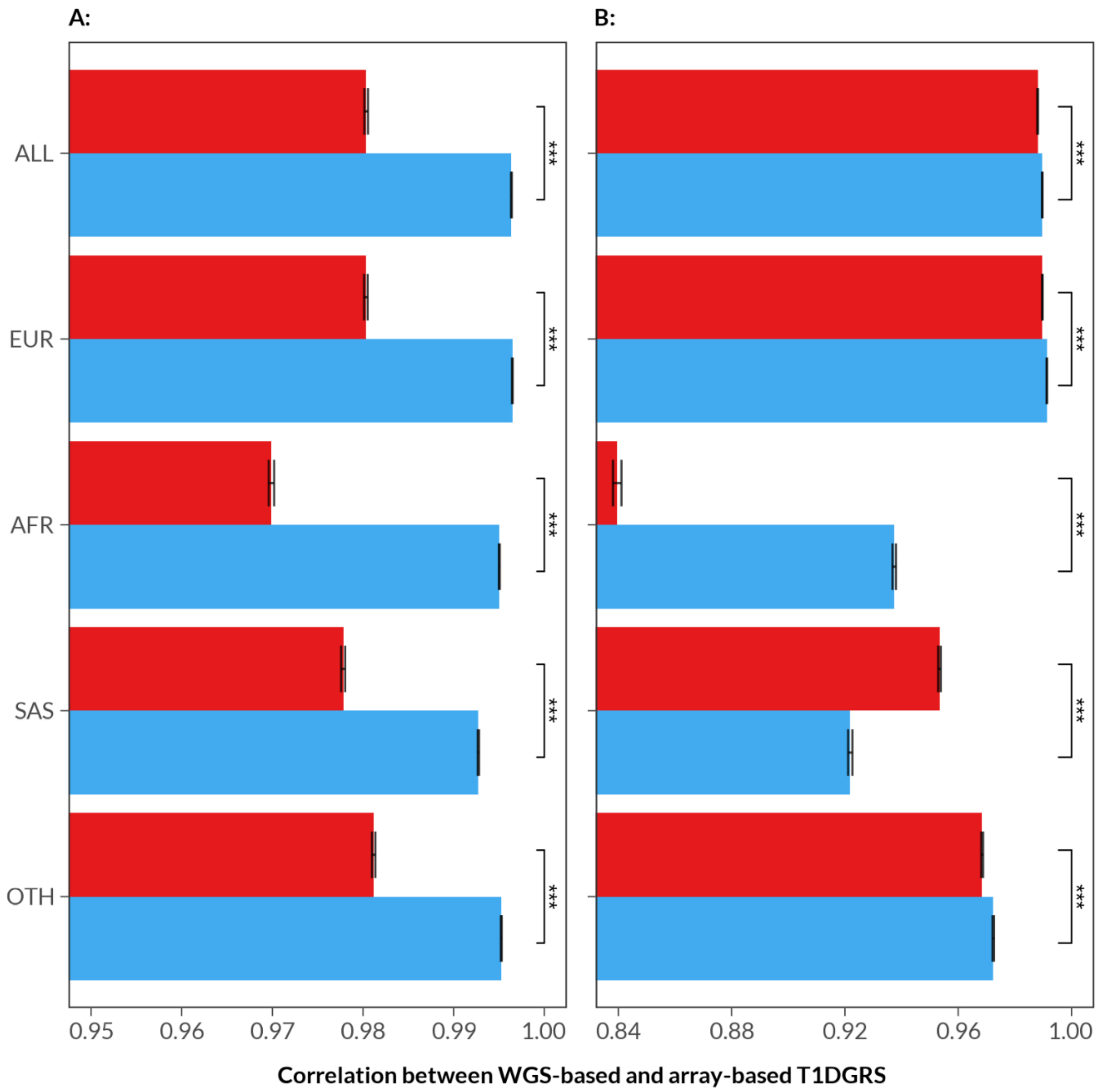

***Supplementary Figure 2: Bar plots showing Spearman's rank correlation between array genotype-derived T1DGRS against WGS-derived T1DGRS** stratified by imputation reference panel, split by effect of variants lying in the HLA region **(A)** and those that are outside it **(B)**. Correlation between WGS-based vs 1000-Genomes imputed array-based T1DGRS is shown in red and between WGS-based vs TOPMed-imputed array-based T1DGRS is shown in blue. Also denotes whether the differences in correlation between corresponding imputation panel pairs are statistically significant, using the correlation z-test by Meng et al. ***p < 0.001 from the correlation z-test (by Meng et al.).*

Supplementary Figure 3

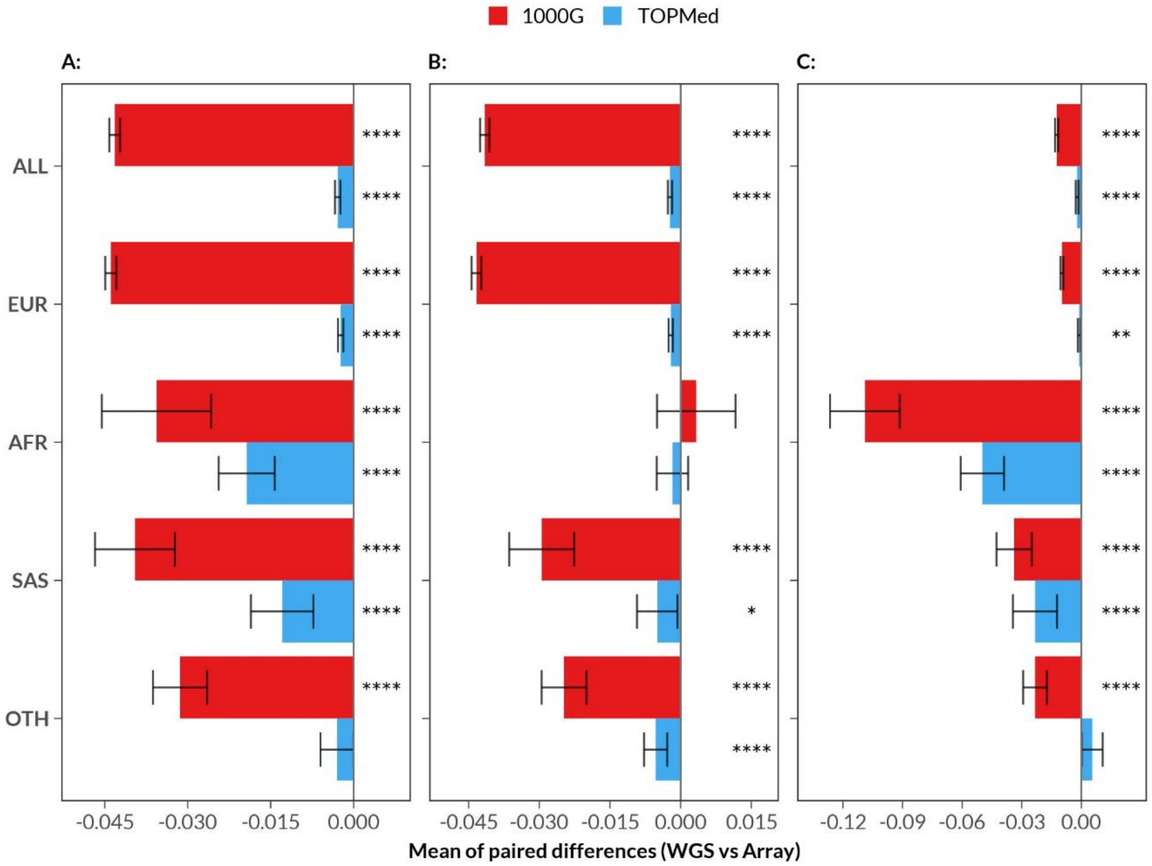

Supplementary Figure 3: Mean difference between WGS-derived T1DGRS and array-derived T1DGRS stratified by genetic ancestry. *Bar graph showing mean differences between WGS and array are calculated for the* ***(A)*** *overall score, and also split by the* ***(B)*** *HLA and* ***(C)*** *non-HLA components of the T1DGRS. (EUR/European, n=137,888; AFR/African, n=2,404; SAS/South Asian, n=3,346; OTH/Others, n=5,627). *p < 0.05, **p < 0.01, ***p < 0.001, **p < 0.0001 from the Bonferroni-corrected paired t-test comparisons between WGS vs array GRS.*

Supplementary Figure 4

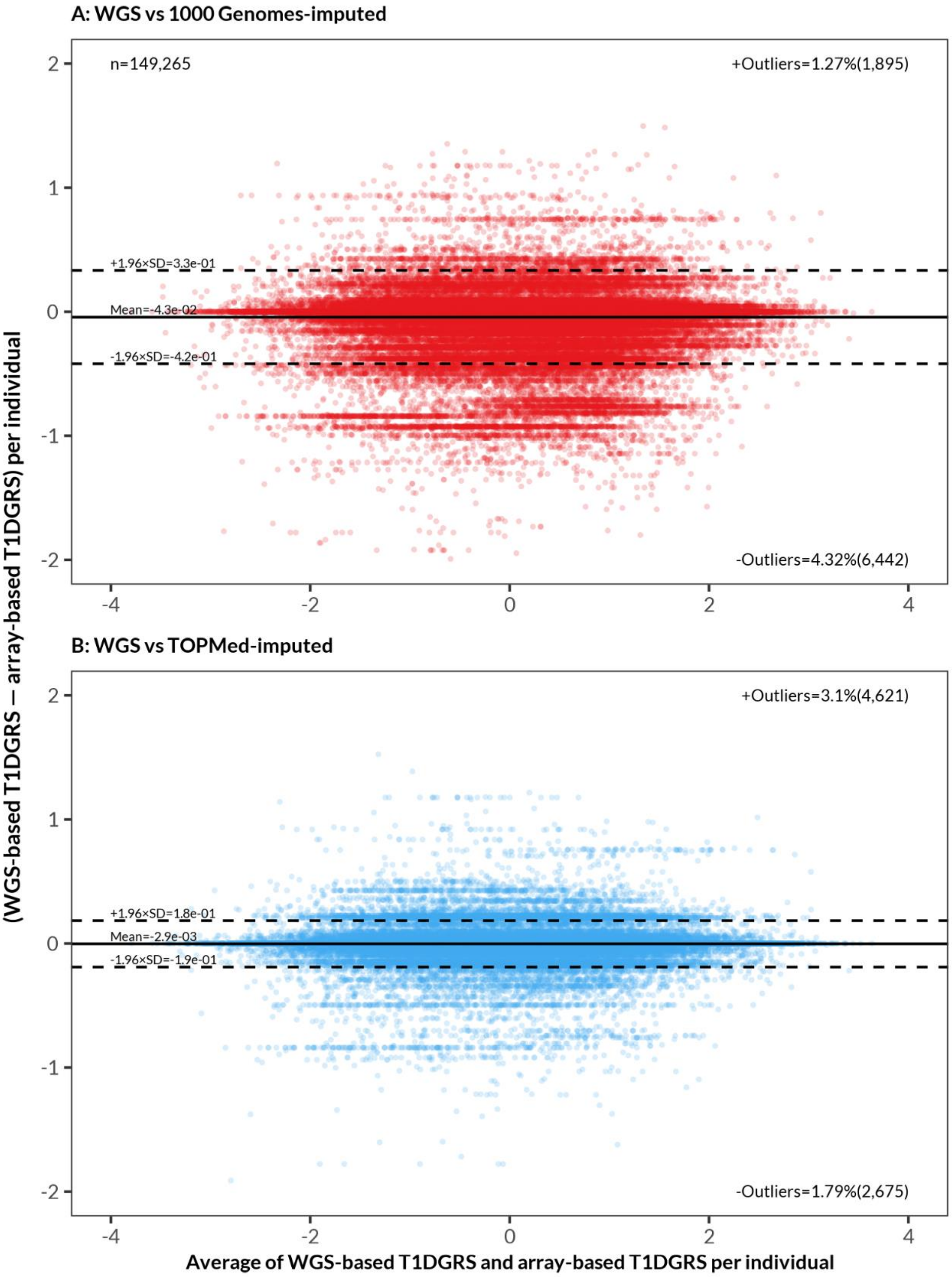

Supplementary Figure 4: Bland-Altman plots showing fixed bias but no proportional bias between (A) WGS-derived vs 1000 Genomes-derived T1DGRS and (B) WGS-derived vs TOPMed-derived T1DGRS, when compared per individual. *The x-axis denotes the average between WGS-based vs array-based T1DGRS per-individual, and the y-axis denotes their differences. Mean of the differences in T1DGRS was found to be ten-fold larger in the 1000 Genomes comparison than in the TOPMed comparison (against WGS), with larger 95% confidence intervals. However, proportion of outliers seem to be about the same in both imputation panels.*

Supplementary Figure 5

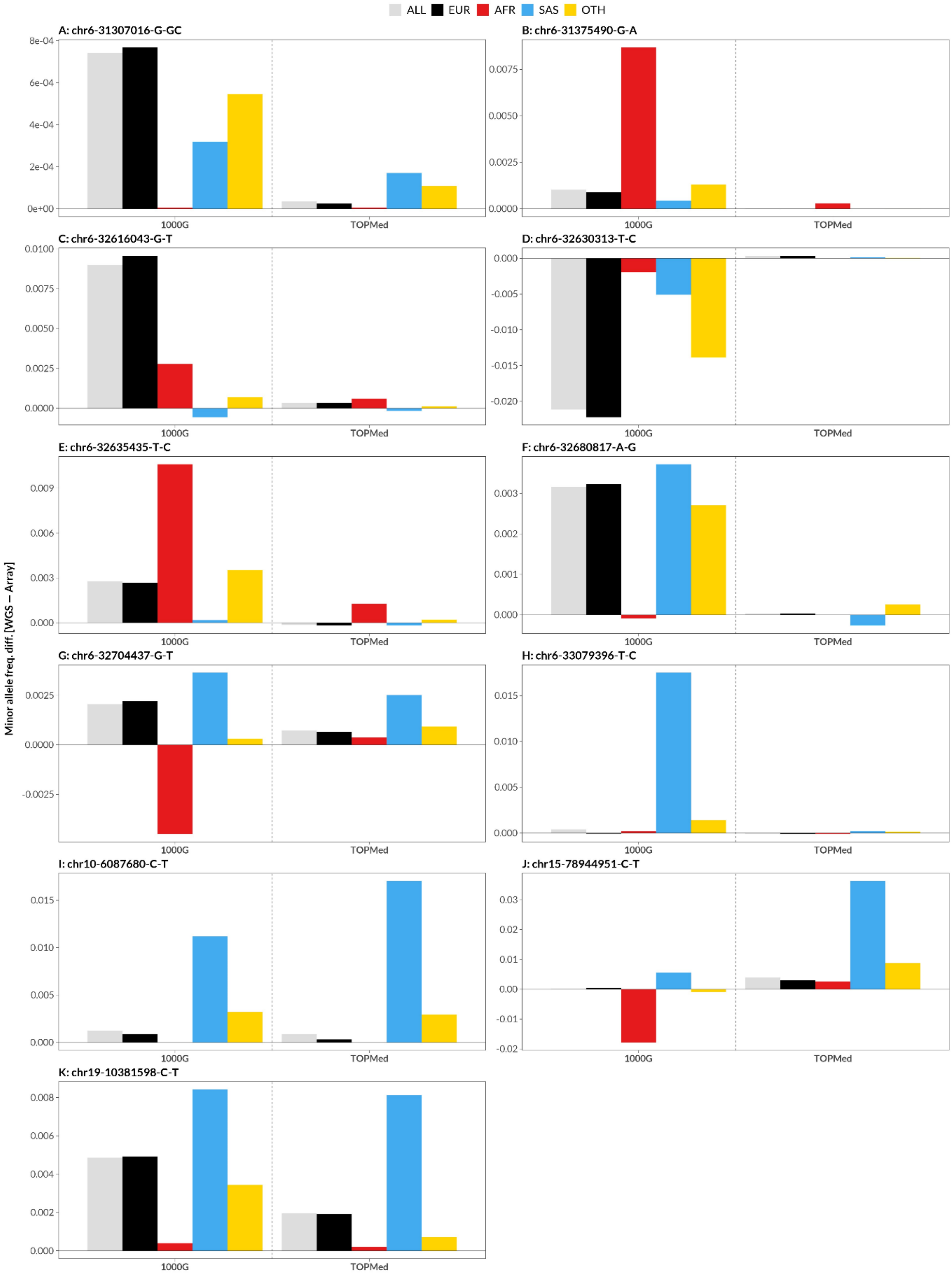

Supplementary Figure 5: Comparison of minor allele frequency differences between variants (GRCh38) in the WGS data and the imputed array genotypes data (i.e., WGS – array) that are Bonferroni significant*, split by imputation reference panel and stratified by genetic ancestry.*

Supplementary Figure 6

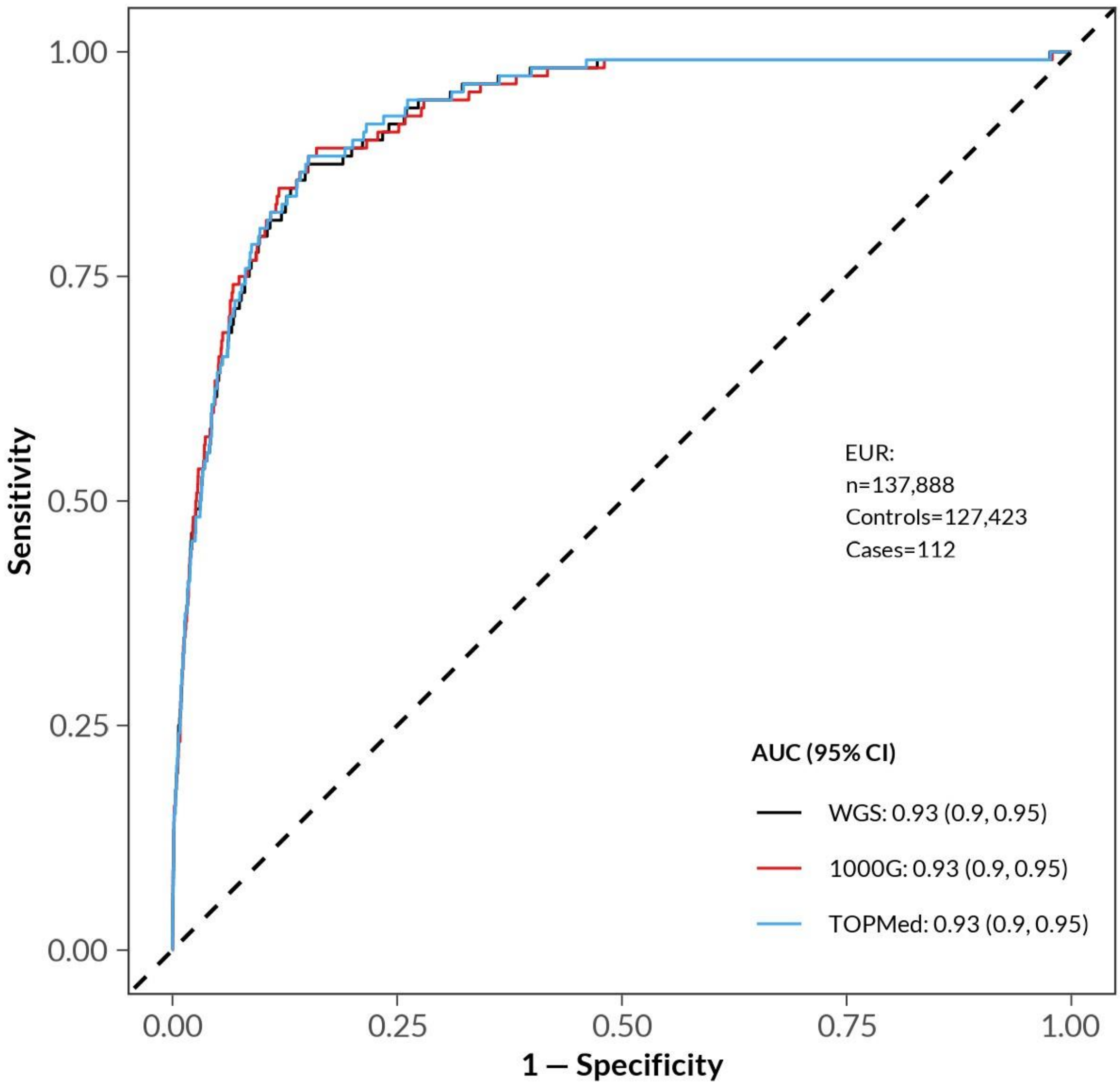

Supplementary Figure 6: Receiver-operator characteristics (ROC) curve analysis to assess the ability of the T1DGRS to discriminate between T1D cases and controls for European individuals (EUR/European, n=137,888), showing nearly identical discrimination across sequencing technologies. *The discriminative ability is quantified using the area under the curve (AUC) with 95% confidence intervals, with AUC = 0.5 achieving case/control discrimination equal to a 50% chance and AUC = 1.0 achieving perfect discrimination.*

Supplementary Figure 7

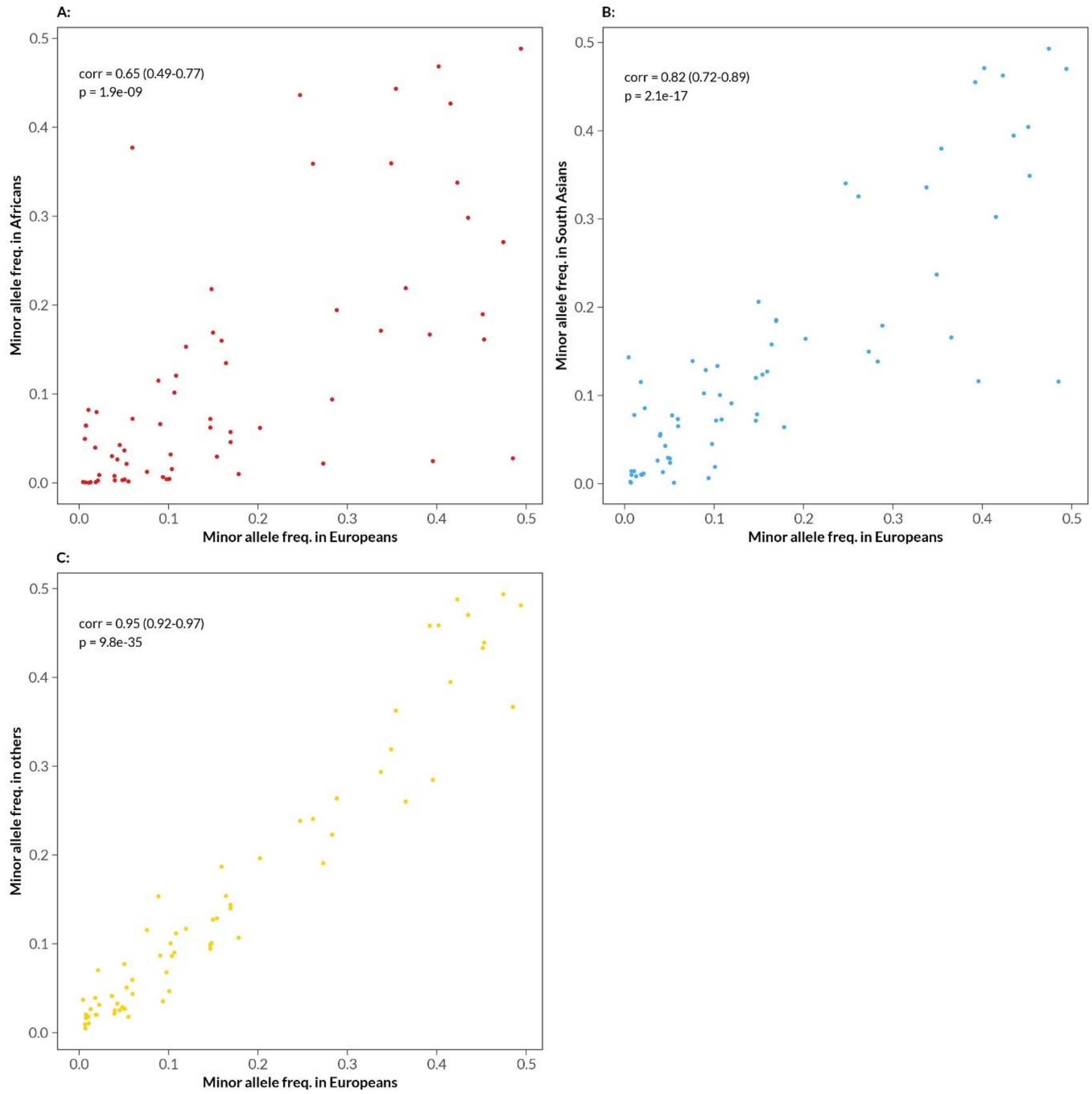

Supplementary Figure 7: Scatter plots showing WGS-derived minor allele frequency comparison between European and (A) African, (B) South Asian and (C) other genetic ancestry individuals *for each variant of the T1DGRS (n=67), with their corresponding correlation coefficients, 95% confidence intervals and p-values.*

Supplementary Figure 8

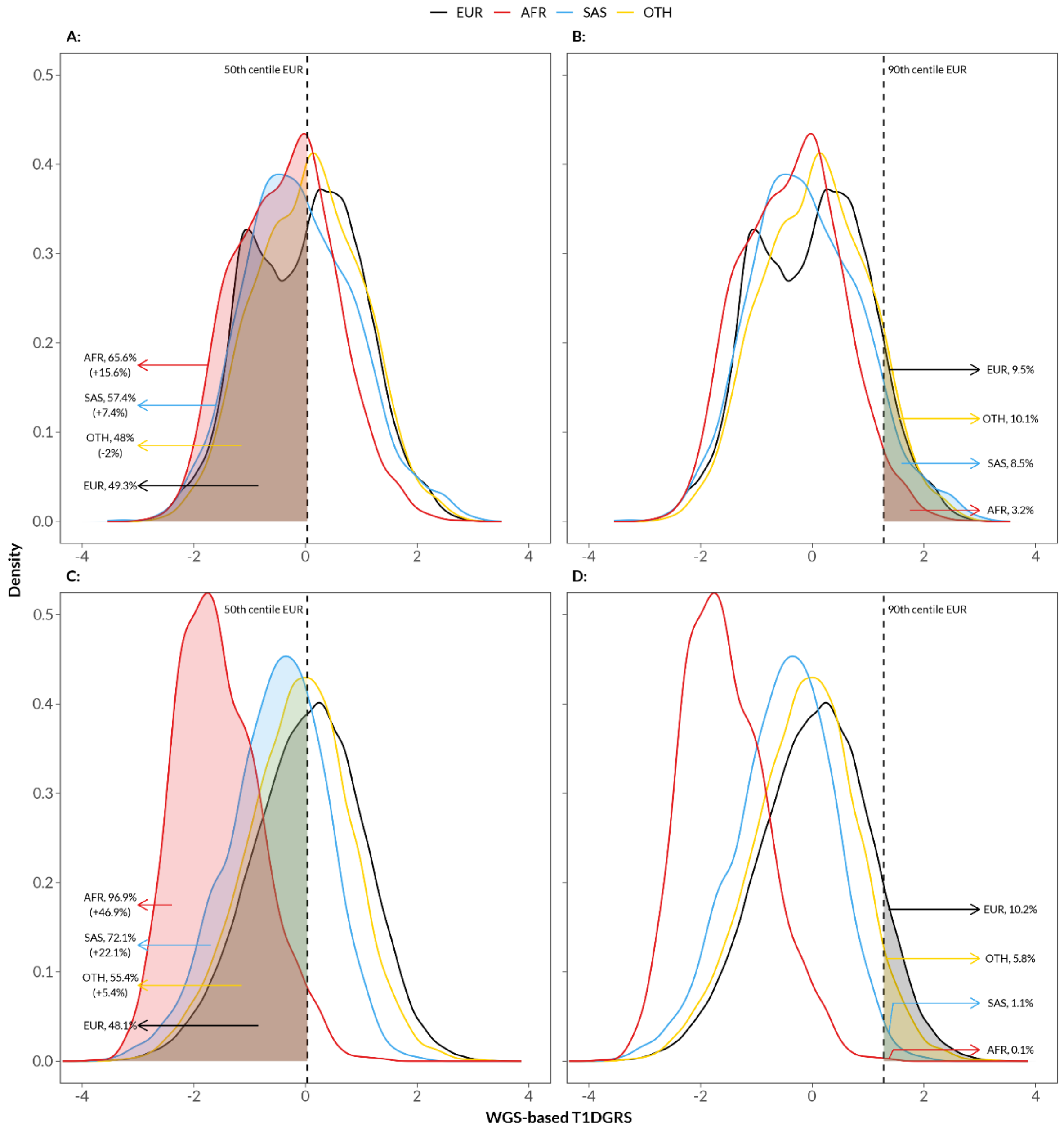

Supplementary Figure 8: Density plots of standardised WGS-derived T1DGRS stratified by genetic ancestry and split by the effect of variants within the HLA region (A and B) and those outside it (C and D) *(EUR/European, n=137,888; African/AFR, n=2,404; SAS/South Asian, n=3,346; OTH/Others, n=5,627). Shows clinically relevant risk thresholds* ***(A and C)*** *50th centile and* ***(B and D)*** *90th centile used in T1D screening processes, calculated based on the overall T1DGRS (i.e., not split by HLA or non-HLA components) of the European genetic ancestry individuals, and the proportion of individuals captured by them.*
